# Reaction of Wheat to European Virulence Races of Common Bunt (*Tilletia* spp.) and Mapping Race-Specific Resistance Genes Using SNP Markers

**DOI:** 10.3390/plants15081264

**Published:** 2026-04-20

**Authors:** Anders Borgen, Dennis Kjær Christensen

**Affiliations:** 1 Agrologica, 9550 Mariager, Denmark; 2 Independent Researcher, 9520 Skørping, Denmark; dennis@fastcode.dk

**Keywords:** common bunt, Bt resistance genes, race-specific resistance, SNP genotyping, marker-assisted selection

## Abstract

Common bunt of wheat (*Tilletia* spp.) remains a significant threat to wheat production in low-input and organic farming systems, where chemical seed treatments are restricted or avoided. Host resistance represents a key component of sustainable disease control, but its effective deployment requires detailed knowledge of race-specific virulence and the genetic basis of resistance. In this study, we analysed the reaction of a large and diverse wheat germplasm collection to current European populations of common bunt and mapped the underlying resistance genes using SNP-based approaches. A total of 2731 wheat accessions were phenotyped from 2012 to 2025 using up to 42 purified bunt races with well-defined virulence profiles. Based on phenotypic responses to race-specific resistance patterns, accessions were grouped and compared with established differential lines. A total of 1504 selected accessions were genotyped using Illumina 26k SNP arrays, and resistance loci were identified by genome-wide association studies followed by fine mapping using recombination analysis. All classical Bt resistance genes from *Bt1* to *Bt10* and *Bt13* and *BtZ* were mapped to defined physical intervals, and the genomic positions of 18 additional race-specific resistance genes were identified in a panel of germplasm. Our results confirm that several historically defined Bt genes, including *Bt11* and *Bt12*, represent multi-gene resistance complexes rather than single loci. Also, genes established as separate genes may possibly be identical, including *Bt4* being identical to *Bt6*, *Bt10* being identical to *BtZ*, and *Bt9* possibly being identical to one of the genes in the *Bt11* complex. These findings highlight the need for a revised nomenclature of genes and a differential set of varieties. The identified resistance haplotypes provide an improved tool for marker-assisted selection and support the development of wheat cultivars with durable resistance to common bunt.

## 1. Introduction

### 1.1. Common Bunt

Common bunt in wheat is an epiphytotic seed-borne disease caused by two closely related species (*Tilletia caries* (DC.) Tul. & C. Tul. syn *T. tritici* (Bjerk.) Wint. and *T. laevis* J. G. Kühn syn. = *T. foetida* (Wallr.) Liro.). Recent research indicates that the two species are actually morphological forms of the same species [1,2,3]. The same race-specific resistance genes control infection in both forms of common bunt and also control the predominantly soil-borne disease dwarf bunt caused by *Tilletia controversa* (Kühn) syn. *T. contraversa* (Kühn) [4].

Wheat production needs control of common bunt. Wheat is and has always been one of the most grown and most significant crops for feed and human consumption, currently covering 20% of the human intake of calories and proteins [5]. Throughout the history of agriculture, common bunt has been considered one of the most significant diseases in wheat to control [6,7,8].

The impact of common bunt on wheat production significantly reduced after the invention of mercury products as a seed treatment in 1913 [9] and the implementation of Uspulun (phenylmercury acetate) by Bayer in 1914. Mercury products effectively controlled the disease for decades in Europe, whereas in less industrialised agrosystems and regions such as Iran and Azerbaijan, common bunt still caused an average yield loss of 30% in the 1990s [10].

In the 1970s and 1980s, the use of mercury for seed treatment was gradually banned and phased out in the EU and replaced by different synthetic fungicides. Probably as a combined result of increased wheat production and a reduced effect of the pesticides replacing mercury-based products, the occurrence of bunt increased in Europe [11]. In organic farming and for other farmers using home-saved seeds without seed treatment, common bunt still causes problems [12].

Infection by common bunt in a seed system multiplies by a factor of 100 from year to year in susceptible varieties [12]; hence, even a small infection in a field will initiate epiphytotic development, and after just a few years, the infection will multiply to a significant level. As few as 100 fungal spores per gram of grain can, in some cases, be sensed by consumers, depending on the age and moisture content of the spores [13], and this level in the grain can be caused by as few as a few infected plants per hectare [12,14]. The accepted threshold for common bunt infestation in seed lots is thus very low, practically at the detection limit of standard seed analysis [15], as even a small infection can reduce grain quality and will inevitably multiply and cause risk for neighbouring fields and the seed system in the region.

Pesticides for seed treatment in modern agriculture provide effective control against common bunt, and significant epiphytotic outbreaks of common bunt almost exclusively occur in untreated seed, whereas dwarf bunt occurs in regions like Sweden, the Alps, and the Pacific Northwest in America, where specific climatic conditions favour soil-borne infections of this disease [16,17,18]. As a result, resistance to common bunt has been a low-priority topic among geneticists and wheat breeders, particularly in Europe, and the disease has been categorised as a “hidden disease” neglected in both farmers’ awareness and in research [18].

The EU and many national authorities are concerned about the widespread use of pesticides in modern agriculture. Fungicides used for the control of common bunt include a group of PFAS-based pesticides, including fludioxonil and sedaxane, and another group containing carboxamide, phenylamide, and strobilurins. Authorities are implementing restrictions on these two groups of fungicides because of their persistence and ecotoxicity in the soil. Therefore, the most widely used group of fungicides in the EU today is triazols, accounting for 95% of annual consumption in Denmark [19]. Triazoles are currently focused on as a risk factor for human health, particularly in regard to the development of fungicide resistance in human medicine used in the treatment of fungal infections such as aspergillosis. The European Farm to Fork and Biodiversity Strategies, therefore, aim to reduce the use and risk of chemical pesticides by 50% before 2030 [20,21]. Little has so far been achieved to meet this goal when it comes to the control of seed-borne diseases.

If common bunt is to be controlled in modern farming with environmental and healthier alternatives to pesticides, there is a range of solutions, such as heat treatment, biological products, and seed analysis combined with discarding contaminated seed lots [22]. In the current article, we focus exclusively on the progress made to use marker assisted selection in plant breeding to develop resistant varieties, with a particular focus on race-specific resistance.

### 1.2. Resistance Mechanisms

Host resistance is a well-known strategy for controlling plant diseases, and resistance to common bunt was among the first breeding goals for breeders and plant pathologists, particularly in Australia and North America [6,7].

As early as 1764, Tschaner observed that different types of spelt (*Triticum spelta*) differed in their susceptibility to common bunt [6], and Kühn also observed, in 1880, varietal differences in susceptibility to bunt in bread wheat (*T. aestivum*) [23]. Already in the beginning of the last century, there was basic knowledge on susceptibility and resistance, and successful breeding programmes were established in the beginning of the century by Farrer [24], Pye [25], and others. Based on studies on phenotypic reactions to different races of bunt, the resistant varieties were grouped according to resistance factors, a fundamental development for the Bt-resistance genes known today.

Early research has shown that inoculating wheat varieties with spores from the same variety often results in higher infection than infecting with spores from other varieties [26,27,28], demonstrating the presence of race-specific resistance in wheat and virulence specialisation in pathogens. Hence, it has been known for about 100 years that race-specific resistance in both pathogen and wheat has a major impact on common bunt infections. Taking virulence into account is pivotal in the use of host resistance as a control strategy for common bunt, as resistant varieties may lose resistance when new races multiply within a region [29].

### 1.3. Genetic Origin of Common Bunt Resistance Genes

Today, the designation of race-specific resistance genes against common bunt is based on the Bt-classification system covering genes *Bt1–Bt7* [30] and later supplemented with the additional genes, including *Bt8* [31], *Bt9* [32], *Bt10* [33], *Bt11* [34], *BtZ* and *Bt12–15* [35], and *BtP* [36].

The Bt genes were defined based on segregation and phenotypic studies, but the development of novel genetic tools, including new knowledge of the genetic position of the genes and genetic markers, has made it possible to study Bt genes and other bunt resistance genes in further detail.

The *Bt1* gene was discovered in the variety ‘Martin’ and therefore previously designated the Martin factor [37]. The resistance of ‘Martin’ later turned out to be caused by a combination of two genes [38], and Briggs and Holton identified and designated them *M*_1_ and *M*_2_ [39]. *M*_1_ was later renamed to *Bt1*, and *M*_2_ was renamed to *Bt7*, proposing Sel 2092 (PI 554101) and Albit as a differential line for *Bt1* [30]. *Bt1* was mapped to chromosome (Chr) 2B in 1960 [40], and a more precise position was only recently presented [41].

On the same chromosome, Chr 2B resistance associated with bunt resistance has been mapped in two different diverse panels of wheat varieties, but it was not discussed whether it was a mapping of a known gene [42,43]. In another study of a diverse set of varieties, the marker Ku_c71357_859 at position 581.70 Mbp on Chr 2B was associated with bunt resistance and suggested to be *Bt1*, as the differential line for this gene, PI 554101, had a positive allele for this marker [44].

McCartney et al. associated resistance in the Canadian spring wheat variety ‘Kenyon’ with Chr 2B and argue that this resistance was a quantitative trait most likely inherited from ‘Neepawa’ and hence different from race-specific Bt genes such as *Bt1* [45]. In another Canadian spring wheat variety ‘CDC Go’, a QTL for resistance was also mapped to Chr 2B at 244 Mbp [46].

The gene *Bt2* was originally found in the variety ‘Hussar’ and designated the ‘Hussar Factor’ or *M*_3_ [47], but this variety turned out to include also *Bt1*, and Metzger designated it *Bt2* and proposed ‘Selection 1403’ and ‘Selection 1075’ as differential lines for *Bt2* [30]. Also, ‘Canus’ is mentioned as an original source of *Bt2* [34]. Goates changed the recommendation to line ‘Selection 1102’ (PI 554097) as a differential line for *Bt2*, but the origin of this line is not described [36].

We previously made a preliminary mapping of *Bt2* from PI 554097 to Chr 1D [48]. On the same chromosome, a QTL, Qcbt.spa-1D, was mapped in ‘Vesper’, but it was characterised as a quantitative trait for bunt resistance and hence different from race-specific Bt genes such as *Bt2* [49].

The gene *Bt3* was first found in varieties ‘Florence’ and ‘Genoa’ developed by Farrer from ‘White Naples’, ‘Improved Fife’, ‘Hornblende’, and an Indian wheat [24,50], and its resistance was studied in further detail by Churchward and Gaines, demonstrating recessive inheritance [51,52]. The variety ‘Ridit’ (Wash. No. 2324, C. I. No. 6703) was developed at Pullman by Gaines in 1915 from a cross between ‘Turkey’ and ‘Florence’ and was registered in 1926 as a bunt-resistant variety [53]. ‘Ridit’ was used as a differential line to separate bunt races already by Bressmann [54], and Metzger designated the resistance *Bt3* with reference to resistance in ‘Florence’ and ‘Ridit’, noting that the chromosomal position was unknown [30].

A resistance factor was found by researchers at BOKU, Austria, and the factor was associated with bunt resistance in a diverse set of varieties with respect to the position 473.96 Mbp at Chr 1A [44]. Later, researchers at the same institute mapped a QTL Qbt.ifa-1AL from the varieties ‘Blizzard’ and ‘Bonneville’ at Chr 1A and hypothesised that this may be the *Bt3* gene [55]. This hypothesis was supported by Lunzer et al. [56].

The gene *Bt4* was originally called the Turkey Factor by Briggs [57] and preliminarily associated with Chr 1B using monosomic analysis [58]. The resistance was originally found in the varieties like ‘Bison’, ‘Kaw’, ‘Nebred’, ‘Omaha’, ‘Oro’ (along with *Bt7*), ‘Turkey 2578’, ‘Turkey 3044’ (along with *Bt7*), and in ‘Turkey 1558’, and resistance is linked with resistance to *Bt6* [59] and also to *Bt5* at Chr 1B [30,60]. ‘Turkey 3055’ was originally proposed as a differential line for *Bt4* [30], but it was later changed to CI 1558 (PI 11610) [35,36].

The *Bt5* gene was originally found in the varieties ‘Hohenheimer Begrante’ (En: awned) and ‘Hohenheimer Unbegrante’ (En: unawned), but it was shown that they were resistant only in Germany but susceptible in Pullmann, USA [61], and the resistance was inherited independently from resistance in ‘Hussar’ (*Bt1* + *Bt2*) [54,62].

It has long been known that ‘Hohenheimer’ (CI. 11458) has not only one but at least two bunt resistance genes [63]. To solve this problem of dual genes in ‘Hohenheimer’ (CI 11458), Hoffmann and Metzger proposed using ‘Selection R60-3432’ (‘Elgin’*’Hohenheimer’) as a differential line for *Bt5* [4]. USDA-NSGC has no record of this accession. Goates returned to ‘Hohenheimer’ (CI 11458) as a differential line [35,36], and despite the documented dual resistance genes, this accession has been used in most studies since. ‘Hohenheimer’ (CI 11458) has a reaction to virulence, demonstrating *Bt5* behaviour but with a lower infection level than most other *Bt5* lines, confirming that an additional and most likely race-non-specific gene seems to contribute to the resistance [64,65].

The *Bt6* gene was originally found in the variety ‘Rio’ and was later identified in ‘Turkey 10095’, ‘Turkey 10097’, and ‘Columbia’ (along with *Bt1*). The gene is also present in ‘Hyslop’ (along with *Bt7*), and resistance was very closely linked to resistance in ‘Turkey’ (*Bt4*), with a recombination value of 2–4% [66]. Later, it was demonstrated by linkage studies and monosomic analyses that the ‘Turkey’ factor T (*Bt4*) and the Rio Factor R(*Bt6*) are linked with the gene that regulates red glume colour on Chr 1B [30,58,60]. Goates tested ‘Rio’ and the other Bt differential lines with 56 global races of common bunt, and five races could separate *Bt5* from *Bt4* and *Bt6*, but none of the races could separate *Bt4* from *Bt6* [36].

#### 1.3.1. Other Resistances Related to Chr 1B

Leijerstam identified European winter wheat ‘Trintella’ as a highly resistant variety [67]. The resistance of ‘Trintella’ was confirmed by Denneken and Pedersen [68], and the dominating resistance factor in ‘Trintella’ was mapped to Chr 1BS [69].

Wang et al. identified a dominating resistance factor in variety ‘Blizzard’ associated with three markers Xgwm374, Xbarc128, and Xgwm264 at a 3,9 cM interval at Chr 1BS [70], and the mapping was later improved to a position at 8–22 Mbp [55]. The authors argue that the ‘Blizzard’ resistance at Chr 1B could be the same resistance found in the Canadian spring wheat variety ‘AC Domain’ on Chr 1BS [71] and in ‘Carberry’ [72] in the same chromosomal region. The mapping of resistance in ‘Carberry’ has been refined to a position at 21.4 Mbp, and it was concluded that it overlaps with resistance from ‘Blizzard’ at Chr 1B [46]. Bunt resistance has also been mapped to Chr 1B between the markers BS00086854_51 and wsnp_Ex_c5679_9976893 in the Canadian spring wheat variety ‘CDC Go’ [73]. An association between marker BS00086854_51 and bunt resistance was confirmed in a diverse set of winter wheat varieties [44], but resistance associated with Chr 1B was not confirmed in a ‘CDC Go’ bi-parental population crossed with ‘Attila’ [46].

A resistance factor was mapped in a diverse panel of varieties to an interval of 137.13–163.10 Mbp on Chr 1B, but it was not discussed whether it was a mapping of a known gene [43].

The *Bt7* gene was discovered as one of two resistance factors, M and M_2_, in the variety ‘Martin’ [39]. The M gene was later renamed to *Bt1*, and M_2_ was renamed to *Bt7* [30]. The Martin factor M_2_ (=*Bt7*) was mapped using nullisomic and monosomic lines to Chr 2D [40]. ‘Selection 50077’ (PI 554100) is a selection from a cross of ’Martin’ with ‘Elgin’ and is now used worldwide as the common bunt differential lines for *Bt7* [35,36].

The *Bt8* gene was originally found in the variety ‘Yayla 305’ (PI 178210). The gene was not mapped, but authors ruled out a position on Chrs 5A, 1B, or 2D [31]. ‘Yayla 305’ is a composite variety developed from local landraces in Turkey and released in 1939 [74]. Later, *Bt8* was also identified in the landrace PI 178383 along with other genes, including *Bt9* and *Bt10* [75,76], and in PI 173438. ‘M72-1250’ (=PI 554120 (PI 173438*Elgin)) was proposed as a differential line for *Bt8* [35,36].

The RAPD marker Psg3 is suggested to link with *Bt8*, but little evidence for this is provided, and information regarding a link to a chromosome or position is absent [77]. The SSR marker Xgwm114 has been used in several studies to assess the presence of both *Bt8*, *Bt10*, and *Bt11* [74,78].

SSR marker wmc112 was linked to bunt resistance on Chr 2D in wheat variety ‘Lewjain’. Segregation demonstrated that a single gene was responsible for resistance. Based on phenotyping with 40 races, *Bt7* and most other Bt genes were ruled out in this variety, but *Bt8* was not [79]. Except for *Bt7*, no other genes have been mapped to Chr 2D.

Bokore et al. contradict the mapping of *Bt8* to Chr 2D with an indication of *Bt8* to be at Chr 7A, but no detailed mapping has been presented [80].

The preliminary mapping of *Bt8* to Chr 7A is not supported in other studies, but many authors have identified resistance to common bunt associated with Chr 7A. Resistance factor Q.DB.ui-7AL derived from resistant line ‘IDO835′ was mapped to Chr 7A [81], and in the highly resistant U.S. sib line of ‘IDO835′, ‘Blizzard’, a QTL Qbt.ifa-7AL for bunt resistance, has been mapped to the position of 722–737 Mbp on Chr 7A, and hence, it is different from *Bt3* and *Bt6*, which are also present in ‘Blizzard’ [55]. Ehn et al. were able to associate marker Ku_c5529_824 at position 335.99 Mbp to bunt resistance in a diverse panel of wheat varieties, and another marker RAC875_c23665_68 was found to be closer to Qbt.ifa-7AL [44]. However, the position of the second marker is contradictory in RefSeg 2.1 [82] and refers to different positions and different chromosomes, including Chr 3A.

In European winter wheat ‘Trintella’, a minor effect of bunt resistance mapped to Chr 7A [69], and also in Canadian spring wheat varieties, ‘AC Domain’ [71], ‘Lillian’ [49], and ‘Carberry’ [81] minor QTLs were mapped to Chr 7A. Virulence to *Bt8* is rare, and given that these mappings are reported as genes with minor effects, it is unlikely that they can express a confirmation of the mapping of *Bt8* to Chr 7A.

Also, at Chr 7A, resistance associated with bunt resistance has been mapped in two different diverse panels of wheat varieties, but it was not discussed whether it was a mapping of *Bt8* or other known genes [42,43].

The *Bt9* gene was originally found in the early 1970s in the landrace ‘CI 7090’ (along with *Bt7*) [32]. The gene has also been found in the landrace PI 178383, along with *Bt10* and other genes [75,76], and ‘Selection M69-2094’ derived from PI 178383*’Elgin’ was used as a differential line for *Bt9* from the mid-1970s [4]. Today, a sib line ‘R63-6968’ (PI 554099) is used as a differential line in most studies [35,36].

Steffan et al. first mapped the *Bt9* gene to the distal end of 6DL using 7k DArT markers [83]. Wang et al. mapped a QTL on 6DL to the interval 469.8–470.3 Mbp in line ‘IDO835’ and hypothesised that this may co-locate with *Bt9* since it possibly is in the pedigree of ‘IDO835’ [81]; this mapping was confirmed and improved by Gordon et al. [84]. Qdb.ssdhui-6DL was mapped later at position 492.5–494.6 Mbp in variety ‘UI Silver’ and was also suggested to be a mapping of *Bt9* [85].

The *Bt10* gene was originally identified in the varieties ‘Greece 18’ (PI 116301) and ‘Mocho’ (PI 116306) [33]. In a screening of virulence in U.S. bunt races, the line ‘M69-2094’, a selection from the cross between ‘Elgin’ and PI 178383, was initially used as a differential line for *Bt10* [4], but it was later changed to the sib line ‘R63-6982’ (PI 554118), widely used today as a differential line for *Bt10* [35,36].

*Bt10* has been mapped to 6DS and was the first Bt gene to be mapped with markers useful for selection [86,87,88]. Cichy and Goates identified RAPD marker 196 to be the best of the three used markers in predicting *Bt10*, with a positive hit rate of 70%. However, the target alleles of RAPD 196 were also present in some genotypes known not to have *Bt10* [77]. ‘AC Cadillac’ is a Canadian spring wheat carrying *Bt10* [89], and Singh et al. mapped a resistance gene to 6D in ‘AC Cadillac’ between flanking markers wPt-672044 and wPt-5114, arguing that this gene is *Bt10* [72]. A refined mapping of *Bt10* resistance in spring wheat ‘Peace’ found the position to be at 7.4–7.6 Mbp [46]. This mapping was further refined in a diverse panel of varieties at 7.43 Mbp and was indicated to be a mapping of the *Bt10* gene [90]. Qdb.ssdhui-6DS was mapped at position 1.4–2.1 Mbp in the variety ‘UI Silver’ and is suggested to be a mapping of *Bt10* [85].

The term *Bt11* was first used by Abdalah for a resistance gene identified in the landrace ‘Dimenit’ along with other resistance genes [34]. This landrace was collected in Tokat, Turkey, in 1948 by V. Taysi at the Turkey’s Field Crops Research Institute and was donated to USDA NSGC by J.R. Harlan, and it was recommended as a new source for breeding [91]. Abdalla describes *Bt11* as a gene in ‘Dimenit’ (PI 166910), in addition to *Bt7* and *Bt9*, also present in the landrace [34]. Goates proposed selection ‘P68-1336-7’ (PI 554098, (‘Elgin’*PI 166910)) as a differential line for *Bt11* [35,36].

In the first attempt for a mapping of *Bt11* in 2011, resistance was associated with Chr 3B and associated with marker loci Xbarc180, Xwmc623, Xwmc808, and Xgwm285, but the author notes that more precise studies of the association are necessary [92]. Cichy and Goates used SSR marker Xgwm114 to identify *Bt11*, and this marker was used in several studies to assess the presence of *Bt11* in wheat varieties [74,78,93]. Resistance associated with Chr 3A has also been demonstrated in a diverse panel of varieties, but without reference to a specific resistance gene [43].

The association of *Bt11* with Chr 3B could not be confirmed in later studies, but the presence of multiple resistance genes on different other chromosomes in the donor of *Bt11* has been identified in several mapping populations [94]. A single QTL, Qbt.ifa-6DL, was found at Chr 6D at 482.8–495.2 Mbp, close to the position of the preliminary mapping of *Bt9* [83]. Since *Bt11*, by the definition given by Abdalla, is a gene additional to *Bt9* [34], it can be speculated that Qbt.ifa-6DL is in fact *Bt9*. However, based on the position of peak markers and of contrasting alleles of the markers within the mapped interval, Lunzer et al. argue that Qbt.ifa-6DL is different from *Bt9* and hence is the *Bt11* gene. On top of the mapped QTL at 6D, two other QTLs were mapped at Chr 4B, one of which, Qbt.ifa-4BL, was mapped to a region between 662.9 and 671.4 Mbp [94].

An additional factor for resistance in ‘Dimenit’ was mapped to Chr 7B at the position 10.1–12.8 Mbp [94]. In the Canadian spring wheat variety ‘McKenzie’, a QTL has also been associated with Chr 7B. This resistance is characterised as a minor factor of resistance [95]. A minor factor of resistance has also been associated with Chr 7B variety in ‘Trintella’ [69].

A resistance factor was mapped in a diverse panel of varieties to a very large interval 18.10–703.15 Mbp on Chr 7B [43], but given the size of this interval, it cannot be concluded if it co-located with the mapping of resistance on Chr 7B in ‘Trintella’, ‘McKenzie’, or others.

On the short arm of Chr 2A, a QTL (QBt.ifa-2A) flanked by markers Tdurum_contig29983_490 and AX-94381641 2A was identified in a single mapping population between ‘Dimenit’ and the moderately resistant variety ‘Mulan’, but it was not presented whether this QTL was inherited from ‘Mulan’ or from ‘Dimenit’ [94]. Also, at Chr 2A, resistance was mapped in Canadian spring wheat ‘Vesper’ to a much higher position at 745.40–746.74 Mbp [49], and therefore, it is most likely different from Qbt.ifa-2A.

It seems contradictory that ‘Dimenit’ has *Bt9* in addition to *Bt11* [34] and, at the same time, that ‘Dimenit’ had only one major resistance gene on Chr 6D [94]. However, the contradiction is not necessarily absolute, since ‘Dimenit’ is a landrace including genetic diversity, and despite being unlikely, the possibility exists that Abdallah may have analysed a selection of ‘Dimenit’ with *Bt9*, whereas Lunzer et al. may have analysed a selection without *Bt9*. Goates analysed the virulence of 56 bunt races and found that only two races were virulent to *Bt11*. One of these was also virulent to *Bt9*, whereas the other were only slightly virulent to *Bt11* but avirulent to *Bt9* [36].

The gene Bt12 was proposed by Goates using PI 119333 as a differential line [35,36]. PI 119333 is a landrace collected in Elazığ, Turkey, in 1937 and recommended as a new source for breeding [91].

Cichy and Goates associated *Bt12* with the marker Xbarc128, demonstrating a prediction of *Bt12* with a positive hit rate of 75%. However, the target alleles of Xbarc128 were also present in some genotypes known not to have *Bt12* [77].

Müllner et al. investigated PI 119333 in detail, concluding that the main factor of resistance QBt.ifa-7DS|Bt12 is caused by a gene within a 4.3 Mbp interval ranging from positions 6.47 to 10.84 Mbp at Chr 7D, with additional resistance identified at Chr 4B [96].

In ‘Blizzard’, resistance has been mapped to a position of 12.5–15.3 Mbp on Chr 7D [96], and this QTL has also been found to be named Q.DB.ui-7DS in ‘IDO444’, a sibling of ‘Blizzard’ [97]. The markers associated with Q.DB.ui-7DS match the markers in the differential line for *Bt12* and PI 173438 [84].

Singh et al. mapped a gene in the Canadian spring wheat variety ‘Carberry’ at Chr 7D [72]. This resistance is considered race-non-specific and hence most likely different from the main factor of *Bt12* described as highly effective. Chen et al. argue that the resistance at Chr 7DS in ‘IDO444’ is different from resistance at Chr 7D found in ‘Carberry’ [97].

The Bt13 gene was first described and studied by Goates, who found it in the variety ‘Thule III’ (PI 181463) [35,36]. The naming of the donor accession ‘Thule III’ is caused by a mistake during transport data from NordGen to USDA NSGC or by a seed mixture accident, since the original variety ‘Thule III’ (NGB6714) is genetically and morphologically very different from PI 181463, showing another phenotypic reaction [98].

*Bt13* has been mapped to Chr 7D [99].

The *Bt14* gene was proposed by Goates for a resistance gene found in the spring durum variety ‘Doubbi’ (CI 13711) [35] and also present in the hexaploid line ‘Selection 186’ (PI 172201) [100]. Goates later omitted the *Bt14* gene from the differential set because of inconsistent reactions of the gene in different environments, particularly regarding spring- and winter-sown trials [36]. Little is known about the physical position of the gene except that it must be on either the A or the B genome. Wang et al. identified QDB.ui-7AL in hexaploid wheat ‘IDO835’ in the position 732.8–736.7 Mbp associated with bunt resistance and pointed to the fact that it was present in differential lines of both ‘Doubbi’ and ‘Carleton’ [81].

The *Bt15* gene was proposed by Goates [35] for a resistance gene found in the spring durum variety ‘Carleton’ (CI 12064). Similarly to *Bt14*, Goates later omitted Bt15 from the differential set because of an inconsistent reaction of the gene in different environments [36].

The *BtP* gene was designated by Metzger, and Goates [36] included the landrace ‘7838’ (PI 173437) in the differential set for bunt resistance. PI 173437 was collected in 1949 in the Hakkâri region in Turkey, a region that has been identified as one of the hot spots of bunt resistance in their landraces [36]. No publications have investigated the genetic background of *BtP* resistance in further detail.

*BtZ* is a term first used by Goates, referring to resistance from variety ‘Zarya’ [35]. This variety was developed in the Soviet Union [101,102,103]. *BtZ* is supposed to be introgressed into *Triticum aestivum* from *Thinopyrum intermedium* via the line ‘Hybrid 599’ (W0480). The cultivar ‘Zarya’ has ‘Hybrid 599’ in its pedigree and is the main source of *BtZ* in European breeding material [104]. ‘Zarya’ has been widely used as a source of bunt resistance, particularly by the German breeder Cultivari, and included in the variety ‘Tilliko’ [105].

#### 1.3.2. Other Identified Resistance Genes Different from the Bt Genes

The designated Bt genes *Bt1*–*Bt13* may, as described above, be associated with chromosomes 1A, 1D, 2A, 2B, 2D, 3B, 4B, 6D, 7A, 7B, and 7D. However, bunt resistance has also been mapped to other chromosomes in different studies and in different varieties.

A QTL for resistance was mapped to Chr 3A in the variety ‘CDC Go’ [73], and in a diverse panel of varieties, resistance has also been associated with Chr 3A [90]. Mourad et al. associated resistance in a diverse panel of varieties with Chr 3A and also with Chr 3B and Chr 5A [43].

In the Canadian spring wheat variety ‘Lillian’, resistance has been mapped to Chr 3D and Chr 5A [49], and in variety ‘Carberry’, resistance was associated with Chr 4D [72].

On Chr 5B, additional alleles for resistance have been mapped in ‘Trintella’ [69].

On Chr 5D, 6A, and 6B, resistance has been identified in a diverse panel of varieties [43], and also on Chr 6A, a study mapped resistance in the Canadian spring wheat ‘Kenyon’ Chr 6A, arguing that resistance was inherited from ‘Neepawa’ [45].

On Chr 7A, resistance has been mapped in U.S. winter wheat varieties ‘Blizzard’ and ‘Bonneville’ [55], ‘Trintella’ [69], ‘AC Domain’ [71], ‘Lillian’ [49], and ‘Carberry’ [81].

Research and breeding for resistance to common bunt have been performed for over 100 years. Varieties with resistance have been developed, but, going through the published literature, the causal genes in both differential lines and in varieties bred for resistance are, to a large extent, unknown. Most research seems fragmented, focusing on single genes in bi-parental populations or diverse panels of varieties with unknown resistance genes, making it difficult to compare findings in different studies. Hence, breeding programs for resistance today still rely on difficult phenotyping with unknown durability. No genes have been cloned, and no markers have been developed that are reliable for either research or breeding to identify causal genes needed to estimate the resistance in the varieties.

In our daily breeding and seed production of wheat for organic farming, we have not only experienced the challenges of preventing common bunt but also seen the potential of resistant varieties and, in particular, varieties with multiple resistance genes.

The aim of the current work is to collate the phenotyping, genotyping, and published information for the dual purpose of improving the practical use of standard markers for marker-assisted resistance breeding and also to prepare mappings for the cloning of causal gene sequences needed to develop reliable diagnostic markers.

## 2. Results

A diverse set of germplasm was collected from gene banks and plant breeders with both known and unknown reactions to bunt, including the differential lines with known Bt genes *Bt1*–*Bt15* plus *BtP* and *BtZ* [36]. The selected lines were used as crossing partners, developing segregating RIL populations covering all described Bt genes, except *Bt14*, *Bt15*, and *BtP*.

Infections in resistant varieties with known resistance genes were tested for true virulence against the gene in question by re-inoculating spores from the varieties onto differential lines and other varieties with similar resistance genes. We found infected plants in all differential lines, and re-inoculation on the host varieties demonstrated true virulence against all Bt genes, except in the differential lines of *Bt9* (PI 554099), *Bt11* (PI 554098), *Bt12* (PI 119333), and *BtP* (PI 173437).

From initial bulk spores collected from farmers and research stations, we managed to purify races by multiplying them on different resistant varieties [65,106]. The purified races were used to phenotype the wheat panel, including a differential set with known resistance genes.

During the period 2012–2025, a total of 2731 wheat accessions were phenotyped with one or more (up to 42) of the purified races of common bunt, resulting in a total of 23,736 field microplots with 50 plants sown in each. Infection level ranged between 0 and 100% in all races, but infection differed depending on the interaction between race virulence and host resistance.

Based on infections in different races, the varieties and breeding lines were grouped in categories with similar patterns of reaction to the different races. Categories including a differential line were postulated to carry the Bt resistance gene. Categories with a reaction not including a differential line were postulated to possess a combination of genes or a new race-specific gene.

Each group of varieties with a Bt resistance gene or with an undescribed resistance gene was analysed for significant association with TG26k SNP markers, and the resistance gene was preliminarily mapped by GWAS, using the total panel of varieties for validation, and afterwards, they were fine-mapped using recombination analysis based on parental information.

Based on these analyses, we were able to map all Bt genes *Bt1*–*B10* and *Bt13* to positions on a physical DNA map (RefSeg 2.1) [82]; data presented in Table 1. From phenotypic categories not including a differential line with a known resistance gene and hence postulated to potentially possess a new unknown race-specific resistance gene, we managed to map additional genes not covered by the standard collection of Bt genes. We compared these mappings with the previous publications listed above to conclude if a new gene has been discovered or can be linked with already known resistance genes or previously described loci (Appendix B). We conclude that 18 new genes have been mapped and that additional genes have been mapped in other studies not found in our panel of germplasm.

Mapping the causal genes in resistant varieties to a physical position is an important step towards the development of marker-assisted breeding (MAS). To further improve the findings for MAS, we selected a set of markers significantly associated with resistance in the GWAS analyses that can be used for MAS. A single or a few markers are rarely enough to identify a gene in a variety because of monomorphic markers in resistant and susceptible varieties. Therefore, haplotypes specific to resistant varieties are listed in Appendix A. Hence, the intervals listed in Table 1 represent the intervals within which the gene is to be found, whereas the haplotypes in Appendix A are markers giving the best hit rate for separating resistant from susceptible varieties in our diverse validation panel of both European and American varieties and landraces.

## 3. Discussion

From the initial collection of spores, we purified races by multiplying them on resistant varieties, and we managed to develop races with virulence to all genes: *Bt1*–*Bt8* and *Bt10*, *BtZ*, and *Bt13*. This means that either virulence to almost all genes has been in the initial spore collection from within Denmark or has developed through mutation during the first 10 years of race purification. Whatever the reason was, it demonstrates that a single race-specific resistance gene is unlikely to be durable as a standalone strategy to control common bunt if widely used in agriculture within a region. As a consequence, three strategies can be used: (1) Many different genes can be used in different varieties in a region to minimise the risk of primary infection into a seed lot; (2) several genes can be stacked into each variety; or (3) variety mixtures or populations of lines with different resistance genes can be mixed to reduce secondary multiplication. Whichever strategy is chosen, alone or in combination, it is pivotal to know the genes available for breeding and to be able to identify and discriminate breeding lines with different resistance genes.

The resistance genes designated *Bt1–10* and the development of differential lines representing the genes have been a solid basis for research in resistance to common bunt. Today, novel genetic tools have been developed that were not available for the pioneers developing the Bt designation system and selecting differential lines. Using these tools to investigate the details of the mapping of the individual genes, a new understanding of the genes has been revealed. Some resistances previously thought to be caused by single genes, such as *Bt11* and *Bt12*, have turned out to be combinations of multiple genes, whereas other genes thought to be different genes have turned out to be most likely identical genes. Different genes with similar phenotypical reactions cause confusion in the statistical analysis of the association between phenotyping and genotyping. We therefore consider it important to specify the original donor of the genes in question and track the heritage to prevent mistakes.

We have in this study focused on race-specific resistance genes, each with either a major effect in avirulent races or no effects in virulent races. For this case, we have chosen the GABIT protocol, as this tool is specifically designed to analyse the interaction between genes and the environment (in our case, different races) rather than analysing the effect at the infection level [116]. We have used the method of the postulation of resistance genes based on discrepancies in phenotypic reactions to different races. This method is less effective in identifying minor race-non-specific genes, as a phenotyping result of medium infection in all races will, in our system, be assessed as susceptible even though the infection level may be lower than the most susceptible varieties. The fact that we have mapped only race-specific genes does not at all mean that only race-specific genes exist in our panel of varieties, but only that our method was not optimal for assessing minor genes. On the other hand, infection level is expected to be proportional to the concentration of virulent spores on the seed, and we believe that some genes in other studies categorised as quantitative traits may, in fact, be race-specific in some cases if a significant proportion of spores in a bulk mixture of spores used for assessment are virulent while others are not.

On top of defining the intervals by markers with statistical association with resistance, we have used recombination mapping, reducing the defined interval to areas demonstrated to be inherited from the resistant parent. This approach has the advantage of finding more precise intervals of the position of the actual gene, but it bears the risk of concluding the final interval by just a few or a single line, therefore introducing potential experimental errors with significant impacts.

### 3.1. Mappings on Chr 1A, Including Bt3

*Bt3* has previously been found on Chr 3A [107]. We mapped the gene to a 4.8 Mbp interval (Table 1). The mapping confirms and improves previous mappings of *Bt3*. Qbt.ifa-1A was mapped in ‘Blizzard’ and considered to be *Bt3*, but no phenotyping with racial differences was provided [55,56]. We tested the same material demonstrating *Bt3* phenotypic behaviour and genotypic similarity with the differential lines and the original donor, and our mapping confirms *Bt3* in ‘Blizzard’ and in the sister-line ‘Bonneville’.

In a previous study of the U.S. wheat line ‘IDO444’, the resistance factor Q.DB.ui-1A was associated with marker Xcfa2129 at 74 cM in Chr 1A [97]. Ehn et al. mapped resistance factor CB-1A to the position of 473.96 Mbp and argued this to be identical to Q.DB.ui-1A [44]. We are convinced that these QTLs are also mappings of *Bt3*. ‘IDO444’ is a zip line of ‘Blizzard’ and ‘Bonneville’, and therefore, all have most likely inherited *Bt3* from their common ancestor ‘Ridit’.

In a diverse panel of varieties from Nebraska, resistance was associated with position 497.93–499.86 Mbp [43], and also in a diverse panel of Canadian spring wheat, resistance was associated with Chr 1A at position 556.87 Mbp [90]. Based on the position, these two mappings could be mappings of *Bt3*.

We mapped a gene *Bt_Mariann_1A* different from *Bt3* at the position of 1.23–10.42 Mbp on Chr 1A in breeding lines, including spelt in the pedigree. We consider this to be a new race-specific gene for bunt resistance. In Canadian spring wheat varieties, two resistance loci have been associated with Chr 1A at positions 13.37–14.03 Mbp and 4.38 Mbp [90]. Given the positions close to *Bt_Mariann_1A*, a common gene could have been involved in these marker–trait associations.

### 3.2. Mappings on Chr 1B, Including Bt4, Bt5, and Bt6

We have fine-mapped both *Bt4* to position 7.4–28.0 Mbp and *Bt6* to position 16.4–28.0 Mbp on Chr 1B (Table 1). This confirms and improves previous mappings [58,59,108,109]. Genes *Bt4* and *Bt6* demonstrated the same phenotypic reaction when exposed to a diverse set of fungal races in both our own phenotypic trials and by others [34,36,64,106]. It cannot be finally concluded if the two genes are actually the same gene, but most indications point in that direction.

In variety ‘Blizzard’, a dominating resistance factor for resistance was identified between markers Xgwm374, Xbarc128, and Xgwm264 on Chr 1B [70], and the mapping was improved to position 8–22 Mbp with a peak at gwm374 and gwm264, but these markers did not match in differential lines for *Bt4*, *Bt5*, or *Bt6* gene [55]. Using our improved mapping of *Bt4* and *Bt6*, we can confirm that Qbt.ifa-1BS was mapped in ‘Blizzard’ [55] and also mapped in ‘Dimenit’ [94], and in a diverse panel of varieties [44], three are most likely mappings of the *Bt4* or *Bt6* gene.

We improved our previous mapping of *Bt5* [110] to a decreased 120.7 Mbp interval on Chr 1B at 163.23–283.93 Mbp, confirming it to be present in our accession of ’Hohenheimer’ (Ci-11458) and in an accession of ‘Hohenheimer’ used at BOKU Austria provided directly to BOKU by Blair Goates (Hermann Burstmayer, pers. Comm.). The haplotype associated with the gene (Appendix A) is also present in an NIL line with *Bt5* developed by James MacKey [117] and in a range of varieties including a number of European commercial varieties, such as ‘Genius’, ‘Promesse’, ‘Globus’, ‘Tommi’, ‘Bill’ ‘WPB Calgary’, ‘Apostel’, ‘Spontan’, ‘Bosporus’, ‘Tillsano’, ‘Initial’, and ‘Ikarus’, with phenotypic reactions similar to the accession of ‘Hohenheimer’ [64,65]. The presence of *Bt5* in European breeding is, in most cases, not an effect of targeted breeding for resistance but is most likely present randomly from the original genetic background, and the original German landrace ‘Hohenheimer’ may also come from the same genetic background. However, when accession CI 11458 of ‘Hohenheimer’ is ordered from USDA NSGC today, a different line is provided with awns and susceptible to all 10 races of bunt used in our standard screening programme. We conclude that some error has occurred and that CI 11458 today is not identical to the original accession of ‘Hohenheimer’ CI 11458 previously described in the literature as a differential line for *Bt5* [35,63]. Despite the dual resistance genes in ‘Hohenheimer’ and the inconsistency in accession CI 11458, we are convinced our mapping is the *Bt5* gene referred to in the original publications defining *Bt5* [54,61,62,63].

The variety ‘Trintella’ has several factors for resistance, including a dominating factor on Chr 1B near the centromere and closest to marker Xgwm273 [69]. The phenotypic reaction demonstrates that it is infected by several races avirulent to *Bt5*. Hence, the resistance in ‘Trintella’ is different from *Bt5*, despite having a resistance gene close to *Bt5*.

Mourad et al. associated resistance with Chr 1B at position 137.13–163.10 Mbp in a diverse panel of varieties [43]. Several genes have been mapped to Chr 1B, and it cannot be concluded whether the close mapping to *Bt5* indicates the involvement of the same genes or not.

Q Cbt.crc-1B.2 has been mapped in ‘AC Domain’ to Chr 1BL and Q Cbt.crc-1B.1 to Chr 1BS [71], and Qcbt.spa-1B was mapped in ‘Carberry’ to Chr 1B [72]. The mapping of resistance in ‘Carberry’ to Chr 1B was improved to the position of 21.4 Mbp [46]. Both Q Cbt.crc-1B.1 and Qcbt.spa-1B are in the vicinity *Bt6* and could therefore be speculated to be *Bt6* [55]. However, ‘AC Domain’ is characterised as only moderately resistant in Canada, and ‘Carberry’ descends from ‘AC Domain’, with no other parental lines with major resistance [71,72]. In a diverse panel of Canadian spring wheat varieties, resistance was associated with Chr 1B at position 21.0–21.4 Mbp; hence, it is close to the resistance mapped in ‘Carberry’ and the ‘AC Domain’. Despite the close chromosomal vicinity with the mapping of *Bt6*, the quantitative behaviour of Q Cbt.crc-1B.1 or Qcbt.spa-1B resistance in Canadian spring wheat varieties demonstrates that these mappings of resistance are different from mappings of the *Bt6* gene, and only the genes occur in the same chromosomal region.

Zou et al. mapped resistance in the Canadian spring wheat variety ‘CDC Go’, which is flanked by markers BS00086854_51 and wsnp_Ex_c5679_9976893 at Chr 1B [73] positioned at 517.23–551.90 Mbp. In a new study of ‘CDC Go’, resistance was not significantly associated with this position [46]. We have also not found race-specific resistance associated with this position at Chr 1B in our panel.

Galaev et al. introgressed dominant resistance into the intercalary region of Chr 1BL in bread wheat from the telomere region in *Ae. Cylindrica* [118]. No phenotypic reaction with different virulence races was presented, and based on the introgression from *Ae. cylindrica*, it cannot be concluded whether this is an already described gene in wheat or a new one.

### 3.3. Mappings on Chr 1D and 2A, Including Bt2

Resistance gene *Bt2* was mapped to a 2.9 Mbp interval at 41.70–44.67 Mbp on Chr 1D (Table 1), confirming and refining our previous mapping [48]. The mapping was confirmed to fit with the differential line PI 554097 and other lines descending from the original donor of *Bt2* ‘Hussar’.

Accession PI 554097 is the globally used differential line for *Bt2* [35,36], but the origin of this line is not described. Kinship analyses and morphological appearances indicate that it is a selection from a cross between ‘Elgin’ and PI 554102 and that PI 554102 is a selection from ‘Hussar’*’Hard Federation’. Despite the unknown origin, we consider PI 554097 as a true carrier of the *Bt2* gene inherited directly from the original donor of *Bt2*.

The QTL Qcbt.spa-1D identified in spring wheat ‘Vesper’ was mapped to Chr 1D [49]. ‘Vesper’ was not included in our mapping panel, but the distinct positions demonstrate that Qcbt.spa-1D is different from *Bt2*. However, the author mapped the gene to Chr 1D using RefSeg 1.0 [119], but the updated RefSeg 2.1 places the linked marker BS00066855_51 on Chr 1B [82] in the vicinity of the mapping of QCbt.dms-1B.2 mapped in ‘CDC Go’ [73].

### 3.4. Mappings on Chr 2A

Qcbt.ifa-2A was mapped in a mapping population of ‘Mulan’ x ‘Dimenit’ between the markers Tdurum_contig29983_490 and AX-94381641 [94]. We fine-mapped this position to the apical end of Chr 2A 0.3–35.09 Mbp (Table 1). From recombination analysis, we conclude that the resistance is inherited from ‘Mulan’ and not from ‘Dimenit’ and hence not a part of the *Bt11* complex defined by the established differential line.

Qcbt.spa-2A was mapped in the Canadian spring wheat ‘Vesper’ to an interval of 745.41–746.75 Mbp at Chr 2A [49]. The mapping is considered distinct from resistance in ‘Mulan’ because of the chromosomal distance of the mappings.

### 3.5. Mappings on Chr 2B, Including Bt1

The *Bt1* gene was mapped to position 799.98–804.81 Mbp at Chr 2B (Table 1), thereby confirming the previous mapping [41].

On the same chromosome, Qcbt.cph-2B has previously been mapped in a diverse panel of varieties, including varieties with *Bt1*, with two markers on each side of our new mapping of *Bt1* [42]. In another study of a diverse panel of varieties, resistance was mapped to the interval 787.82–785.91 Mbp, slightly beneath our mapping of *Bt1* [43]. Ehn et al. associated marker Ku_c71357_859 at the position of 581.70 Mbp with bunt resistance and suggested that this could be *Bt1* [44]. The genetic position of these mappings in the vicinity of our mapping supports that these resistance–marker associations could indeed be caused by the presence of *Bt1*.

Q.DB.ui-2B was mapped in ‘IDO444’ [97]. The position associated with Xwmc317 at 14 cM and the parental information of ‘IDO444’ indicate that this is different from *Bt1*.

In the Canadian spring wheat variety ‘CDC Go’, a QTL was also mapped to Chr 2B at 244 Mbp and is associated with bunt resistance [46]. The position and phenotypic behaviour indicate that it is different from *Bt1*.

The varieties ‘Hereward’, ‘Bussard’, ‘Skotte’, ‘Complet’, ‘Paroli’, and several other well-known varieties used in European breeding over the past century have been shown to have a phenotypic reaction similar to *Bt2* [64,106]. Genotypic analysis of these varieties from the European genepool reveals that they have not inherited the *Bt2* gene from ‘Hussar’ mapped to Chr 1D but have another race-specific resistance gene at Chr 2B. We mapped this resistance, *Bt_Bussard_2B* in ‘Bussard’, ‘Hereward’, and other European varieties, to 8.70–13.12 Mbp at Chr 2B. The difference in position and phenotypic behaviour demonstrates it to be different from *Bt1* and other resistance genes identified on Chr 2B.

### 3.6. Mappings on Chr 2D, Including Bt7

We have mapped *Bt7* to position 616.02–621.07 Mbp at Chr 2D (Table 1), confirming and refining previous mappings [40,111]. The phenotypic reaction and presence of associated markers demonstrate that *Bt7* is present in a range of commercial varieties of both spring wheat and winter wheat in Europe, including ‘Tambor’, ‘Korrund’, ‘Xenos’, ‘Segor’, ‘Quarna’, ‘Fiorina’, ‘Thomaro’, and ‘Sailor’ [64,106,111]. No other Bt genes have been mapped to Chr 2D.

Bunt resistance in ‘Lewjain’ was shown to be caused by a single major gene linked to SSR marker wmc112 on Chr 2D, but based on phenotyping with different races, the authors argued that this resistance is different from *Bt7* but could possibly be *Bt8* [79]. We have not analysed ‘Lewjain’, but our mapping of *Bt8* to Chr 4A (see below) can disconfirm the author’s speculation that the resistance in ‘Lewjain’ is attributable to *Bt8*. As no other genes have been mapped on Chr 2D and excluding *Bt7*, ‘Lewjain’ may have a hitherto unknown strong gene yet to be studied in further detail.

### 3.7. Mappings on Chr 3A, 3B, and 3D

None of the Bt genes were mapped to Chr 3A, but in the variety ‘Stephens’, we mapped two genes: one on Chr 3A at the position 683.12–688.69 Mbp (Table 1) and a second on Chr 7B (see below). This confirms previous findings pointing to ‘Stephens’ carrying one or more resistance genes effective against a few races [63]. In a diverse panel of Canadian spring wheat varieties, resistance was mapped to Chr 3A at position 671.29 Mbp [90]. Given that resistance in ‘Stephens’ and in Canadian spring wheat is described as involving minor additive genes and due to the close vicinity of the mappings, it is possible that our mapping of *Bt_Stephens_3A* is the same gene associated with resistance in Canadian spring wheat. In the same study, additional resistance was associated with Chr 3A at position 10.28 Mbp [90]. In another study of a diverse panel of varieties, resistance was associated with positions 51.29–53.74 Mbp and also with a wide interval of 69.957–742.47 Mbp [43]. The first position at Chr 3AS is definitely different from *Bt_Spephens_3A*, but given the wide span of the latter, it cannot be excluded from co-locating with our mapping of resistance in ‘Stephens’.

Zou et al. mapped resistance in the Canadian spring wheat variety ‘CDC Go’ associated with markers RAC875_c17453_896 and RAC875_c57584_240 at Chr 3A [73]. This corresponds to position 1.48–251.79 Mbp [82]. It is difficult to interpret if this wide span between the markers is an interval or two different resistance factors, but a re-mapping of ‘CDC Go’ could not confirm any resistance significantly associated with Chr 3A [46].

In a diverse panel of varieties, resistance was mapped to Chr 3B [43], and in the variety ‘Lillian’, resistance was mapped to Chr 3D [49]. We found no resistance in our panel associated with Chr 3B or 3D, and we could not therefore correlate this finding with other genes.

### 3.8. Mappings on Chr 4A, Including Bt8

We mapped *Bt8* close to the end of Chr 4AS below 16.86 Mbp (Table 1).

*Bt8* was originally found in the Turkish mixed line ‘Yayla-305’ [31], but the differential line used for *Bt8* today is PI 554120, descending from landrace PI 173438 [35] and also present in landrace PI 178383, which is extensively used in U.S. wheat breeding. We can confirm that the haplotype recommended for MAS (Appendix A) is present in ‘Yayla-305’ in PI 554120, PI 1 173438, and PI 178383, confirming that the same *Bt8* gene is present in all three sources of *Bt8* resistance. No other studies have mapped bunt resistance genes on Chr 7A.

Our mapping of *Bt8* to a position at Chr 4AS contradicts the findings by Bokore et al. proposing *Bt8* to be at 7A [80], and it also disconfirms the hypothesis that the mapping of a single dominating resistance gene linked to Wmc112 on 2D in the variety ‘Lewjain’ co-locates with *Bt8* [79]. This, however, does not rule out the presence of *Bt8* in ‘Lewjain’, as the marker may have been positioned on the wrong chromosome.

### 3.9. Mappings on Chr 4B, Including Genes Associated with Bt11 and Bt12

As described in the introduction, the donors and differential lines of *Bt11* and *Bt12* each include several bunt resistance genes, and it is therefore debatable which of them should be selected as *Bt11* and *Bt12*, if any.

The landrace ‘Dimenit’ is the donor of *Bt11* in the differential line PI 554098, and Qbt.ifa-4BL was mapped in ‘Dimenit’ [94]. We re-mapped this gene to a position at 657.89–662.87 Mbp (Table 1). This gene is not present in the differential line PI 554098.

Qcbt.ifa-4BS was identified as a resistance factor in ‘Dimenit’ on Chr 4B, but no position was presented [94]. We mapped this gene to 15.74–17.79 Mbp, confirming that it is distinct from Qbt.ifa-4BL. Qcbt.ifa-4BS is present in differential line PI 554098, hence contributing to the excellent resistance of this line.

Also, the differential line for *Bt12*, PI 119333, includes several bunt resistance genes, making it debatable which should represent *Bt12*. Qcbt.ifa-4B was mapped in PI 119333 to a very large interval, covering most of the chromosome at 20.6–706.5 Mbp [96]. We investigated this in further detail and mapped two different genes at opposite ends of Chr 4B: *Bt_PI119333_4BL* at 650.38–670.63 Mbp and *Bt_PI119333_4BS* at 1.31–15.86 Mbp. We found virulence in our spore collection with respect to both of these resistance genes.

Both ‘Dimenit’ and PI 119333 have resistance genes in each of the opposite ends of Chr 4B mapped to overlapping intervals. The haplotype developed for MAS differs, but this could be due to the different donors of origin affecting the population structure of the markers. The genes have not been isolated into distinct lines with only a single gene, and we have therefore not been able to test them with different virulence types with respect to whether they demonstrate identical phenotypic reactions. We therefore cannot at present conclude if there are two, three, or four genes involved in the *Bt11*/*Bt12* complex at Chr 4B.

Singh et al. also mapped Qcbt.spa-4B, conferring common bunt resistance to a region on the short arm of Chr 4B in ‘Carberry’ [72]. This mapping was flanked by wPt-744434–wPt-617, which does not overlap with our mapping, and hence, it is most likely different from the genes mapped in ‘Dimenit’ and in PI 119333.

### 3.10. Mappings on Chr 5A, 5B, 5D, 6A, and 6D

None of the Bt genes was mapped to Chr 5A, 5B, or 5D, but new genes were mapped to these chromosomes.

In the original donor of *Bt8* ‘Yayla 305’, we identified two additional genes different from the *Bt8* gene that were not found in the differential line for *Bt8* PI 554120.

*Bt_Yayla305_5A* was mapped at 572.16–597.23 Mbp on Chr 5A (Table 1), demonstrating race-specific behaviour. In a diverse panel of varieties, resistance was mapped to Chr 5A: 568.05–613.55 Mbp [43]. In the spring wheat variety ‘Lillian’, resistance was mapped to Chr 5A at 659.01–658.82 Mbp, in addition to resistance at Chr 7A (see below) [49]. Given the close mappings in these studies, it is possible that these are mappings of the same gene.

We mapped an additional gene, *Bt_Yayla305_5B*, in ‘Yayla 305’ on Chr 5B at a large interval of 36.90–324.49 Mbp (Table 1). Resistance in variety ‘Trintella’ was associated with marker Xgwm408 at Chr 5B, but genotyping demonstrates that ‘Trintella’ does not have *Bt_Yayla305_5B*.

We did not find any genes on Chromosome 5D, 6A, or 6B and could not therefore confirm or disconfirm mappings published with respect to these three chromosomes [43,45,90].

### 3.11. Mappings on Chr 6D, Including Bt9, Bt 10, and Bt11

We mapped *Bt9* to the distal end of Chr 6D above 490.7 Mbp, confirming and refining previous mapping [83,115]. This finding supports the hypothesis that resistance mapped to Chr 6D in ‘IDO835’ [81,84] and in ‘UI Silver’ [85] is indeed a mapping of the *Bt9* gene.

PI 178383 has been widely used as a donor of *Bt9* in both the USA and Europe, and *Bt9* is confirmed to be present in varieties such as ‘Stava’, ‘Hallfreda’, and ‘SW Magnifik’. Using our proposed haplotype for MAS (Appendix A), we can confirm that the gene is also present in the original donor of *Bt9* ‘CI 7090’, confirming that the *Bt9* genes found in PI 178383 are identical to the *Bt9* originating from the original *Bt9*-donor ‘CI 7090’.

Qdb.ssdhui-6DL was mapped in ‘UI Silver’ to a position at 492.5–494.6 Mbp [85], and we improved the mapping to a position from 492.57 Mbp to the end of the chromosome. Based on parental information and position, we believe that this is an additional mapping of *Bt9*.

Virulence against *Bt9* is present in the USA [97]. Infections in the differential line of *Bt9* are rarely reported in Europe [120,121], but low infections in the differential line for *Bt9* are seen in some trials [56,65,122]. When infected spikes of breeding lines or varieties with *Bt9* were observed in our nursery, spores were used to re-inoculate differential lines with *Bt9* to investigate if infection was a sign of emerging virulence. We also acquired infected plants with *Bt9* from other European countries, but we never managed to confirm virulence or maintain the disease on a differential line with *Bt9* from spores from any of these infected plants. On this basis, we consider it questionable to what extent virulence to *Bt9* is present in Europe.

In the original donor of *Bt11*, the landrace ‘Dimenit’, a major factor for resistance, Qbt.ifa-6DL, has been mapped to Chr 6D at 482.8–495.2 Mbp on top of the resistances mapped on Chr 4B [94]. We mapped this resistance in the differential line of *Bt11*, PI 554098, and in ‘Dimenit’ to an interval identical to that of *Bt9* using haplotype recombination mapping. The question still remains whether *Bt9* and Qbt.ifa-6DL from ‘Dimenit’ are the same genes. Qbt.ifa-6DL has not been isolated into a line without additional bunt resistance genes on Chr 4B, and no virulence against *Bt9* has been found in our spore collection, enabling us to compare the phenotypic reaction of *Bt9* and Qbt.ifa-6DL. Only one race (D-19) has been demonstrated to be virulent to *Bt11*, and this race is also virulent to *Bt9* [36]. This strongly indicates that Qbt.ifa-6DL is in fact *Bt9*, but we still cannot, on the current basis, conclude so.

We mapped *Bt10* to Chr 6D at position 2.05–3.34 Mbp (Table 1), confirming and refining the previous mapping [72,86,87,88,113]. The recommended haplotype for MAS (Appendix A) confirms *Bt10* to be present in PI 178383 and also confirms it to be present in the original donors of *Bt10* ‘Greece 18’ (PI 116301) and ‘Mocho’ (PI 116306) [33]. Given the parental information available and the position of the mappings, we can confirm that the mapping of resistance factor Qdb.ssdhui-6DS in ‘UI Silver’ [85] and the mapping of Qcbt.spa-6D in ‘AC Cadillac’ [72], ‘IDO835’ [84], and Qcbt.dms-6D in both ‘Peace’ [46] and in a diverse panel of Canadian spring wheat varieties [90] co-locate with our mapping of *Bt10.*

The term *BtZ* was introduced by Goates, referring to resistance in the variety ‘Zarya’ [35]. We have mapped *BtZ* to an interval identical to the mapping of *Bt10* (Table 1). Indeed, *BtZ* resembles *Bt10* in many respects, including identical phenotypical reactions to all tested fungal races. If *BtZ* is caused by an introgression from *Thinopyrum intermedium*, we would expect failing markers on the standard TG26k chip in the vicinity of the *BtZ* gene, indicating the alien origin compared with the standard genetic setup from bread wheat, but this has not been observed. We therefore conclude that bunt resistance in ‘Zarya’ is caused by a source in the pedigree other than introgression ‘Hybrid 599’. We cannot finally conclude whether *BtZ* and *Bt10* are identical genes or two different genes.

### 3.12. Mappings on Chr 7A

‘Quebon’ is a French winter wheat with broad resistance to common bunt [122], including *Bt5*. We mapped *Bt_Quebon_7A* to Chr 7A position 671.34–676.63 Mbp (Table 1). *Bt_Quebon_7A* has a race-specific behaviour similar to *Bt2* and *Bt_Bussard_2B*.

Qcbt.spa-7A was mapped in the Canadian spring wheat in ‘Lillian’ to a position at 693.40–598.87 Mbp on Chr 7A [49], close to Q Cbt.crc-7A mapped in ‘AC Domain’ [71]. Both ‘Lillian’ and ‘AC Domain’, as well as the closely related ‘Neepawa’ and ‘Thatcher’, are known to have a quantitative type of resistance. Virulence to *Bt2* was found in 80% of U.S. bunt races, a frequency much higher than virulence to other Bt genes [123]. Testing varieties with a mixture of spores, including both virulent and avirulent spores, for a given gene can produce phenotypic results resembling those of race-specific genes with reduced infection across all races, similar to quantitative genes. The presence of virulence to *Bt2* in Canada may explain the quantitative behaviour of resistance in Canadian spring wheat varieties. Therefore, it cannot be finally excluded that *Bt_Quebon_7A* could be involved in resistance in Canadian spring wheat, which is described to demonstrate quantitative behaviour.

Qbt.ifa-7AL was mapped in ‘Blizzard’ at position 722–737 Mbp [55], and QDB.ui-7AL was mapped in ‘IDO835’ to a position from 732.05 to 736.56 Mbp [81]. We have refined the mapping in ‘Blizzard’ to 717.92–735.89 Mbp (Table 1), confirming that it is distinct from *Bt_Quebon_7A*.

Given the fact that three genes, including *Bt2* at Chr 1D, *Bt_Bussard_2B* at Chr 2B, and *Bt_Quebon_7A* at Chr 7A, produce the same phenotypic reaction similar to *Bt2* and are widely distributed in European winter wheat breeding, there may have been selection pressure on the pathogen towards virulence against these genes in Europe. It is confirmed that half of the races collected in Europe have virulence against *Bt2*, a frequency far higher than virulence to other known Bt genes [65].

In three different diverse panels of varieties, resistances have been mapped at Chr 7A to positions at 444.4 Mbp [42], 298.99 Mbp [43], and 335.99 Mbp and 633.77 Mbp [44]. Also, in ‘Trintella’, resistance has been associated with Chr 7A [69]. Resistance associated with single markers with different marker systems, particularly in diversity panels, is difficult to evaluate and compare, but the systematic findings of resistance associated with Chr 7A may indicate some similarities that could refer to a single or a few common genes widely distributed in the gene pool of modern varieties.

### 3.13. Mappings on Chr 7B

Qbt.ifa-7B was identified as an additional resistance factor in ‘Dimenit’ (PI 166910) on Chr 7B at 10.07–12.80 Mbp [94]. We have confirmed this mapping and conclude that it is not present in the differential line for *Bt11* (PI 554098); hence, it is not involved in the *Bt11* gene complex.

We also mapped another gene on Chr 7B from variety ‘Stephens’ at 630.88–638.50 Mbp. Given the genetic chromosomal distance, we consider this resistance distinct from Qbt.ifa-7B. Resistance in ‘Trintella’ has also been associated with 7B [69], but genotyping demonstrates that ‘Trintella’ does not have resistance from ‘Stephens’.

Qcbt.spa.-7B.1 was mapped in spring wheat variety ‘McKenzie’ and is associated with bunt resistance with peak markers Xgwm573 and Xwmc17 on Chr 7B [95]. In a diverse panel of varieties, resistance was associated with Chr 7B at position 703.15 Mbp [43]. Given the use of different marker systems and uncertain positions, it cannot be concluded whether these mappings refer to the same resistance genes as listed above.

### 3.14. Mappings on Chr 7D, Including Bt12 and Bt13

PI 119333 is the differential line representing *Bt12* [35], and Qbt.ifa-7DS was mapped in PI 119333 to the interval 6.47–10.84 Mbp [96]. We have mapped several genes in PI 119333, including *Bt7* and two different genes at Chr 4B (see above). Our mapping of Qbt.ifa-7DS indicates a slightly elevated position at 14.62–20.93 Mbp (Table 1). Within lines with this chromosomal interval at Chr 7D inherited from PI 119333, we found three different virulence patterns, only one of which confers total immunity. We therefore conclude that the mapped interval at Chr 7D includes two different and independent resistance genes, *Bt_PI119333_7D-1* and *Bt_PI119333_7D-2*, and that total immunity to all races in our collection only occurs when both genes are expressed. Summing up, we have mapped five different race-specific genes in the differential line for *Bt12*, PI 119333.

‘Blizzard’ is another variety carrying multiple resistance genes, including resistance mapped to Chr 7D at 12.5–15.3 Mbp [96]. In wheat line ‘IDO444’, a resistance factor Q.DB.ui-7DS has been mapped to position 5.88 Mbp, and this is likely a mapping of the same gene, as ‘IDO444’ is a sibling of ‘Blizzard’ [97]. Chen et al. argue that Q.DB.ui-7DS fits with the differential line for *Bt12* [97].

We mapped the resistance in ‘Blizzard’ to the position on Chr 7D at 16.71–21.84 Mbp (Table 1), overlapping with *Bt_PI119333_7D-1* and *Bt_PI119333_7D-2*, but there are different haplotypes for lines with different origins of resistance (Appendix A). Therefore, we were still unable to definitely determine whether the Chr 7D resistance mapped in ‘Blizzard’ and ‘IDO444’ is identical to one of the resistance genes mapped to Chr 7D in PI 119333.

The *Bt13* gene was mapped to an interval beneath resistance from ‘Blizzard’ and PI 119333 at 5.01–9.64 Mbp on Chr 7D (Table 1), confirming previous mapping [99]. The phenotypical reaction and the chromosomal distance demonstrate that *Bt13* is different from the genes at Chr 7D found in ‘Blizzard’ and PI 119333.

A resistance factor Qcbt.spa-7D gave a minor effect on bunt incidence in Canadian spring wheat variety ‘Carberry’ and was mapped to Chr 7D flanked by X664136 and Xwmc273 [72]. Resistance associated with Chr 7D in ‘Carberry’ was not confirmed in a later study [46]. The *Bt13* gene and the genes on Chr 7D in PI 119333 demonstrate a clear race-specific behaviour, and the chromosomal distance demonstrates that Qcbt.spa-7D mapped in ‘Carberry’ is different from the resistance factor from our mapping.

In a diverse panel of varieties, a single marker on Chr 7D at 595.91 Mbp was associated with resistance [43], but given its position, there is no indication of co-location with *Bt13* or resistance from ‘Blizzard’ or PI 119333 at Chr 7D.

### 3.15. BtP

The landrace ‘7838’ (PI 173437) is included as a differential line to represent *BtP* [36]. We have not studied segregating RILs of crosses with PI 173437, nor have we found literature references studying *BtP* in any detail. The haplotypes recommended for the MAS of other genes described in this article (Appendix A) indicate that *Bt2* and *Bt7* may be present in PI 173437. Goates studied 56 bunt races and found 12 races virulent to PI 173437. All 12 also had virulence to both *Bt2* and *Bt7*, but many other races virulent to both *Bt2* and *Bt7* were avirulent to PI 173437 [36]. In our own phenotyping, none of the races were virulent against PI 173437, but several had virulence to the combination of *Bt2* and *Bt7*. This indicates, but does not definitely prove, that PI 173437 has a combination of *Bt2*, *Bt7*, and one or more additional known or unknown genes. The term *BtP* as an expression of a specific gene must therefore be used with caution and not be confused with the expression of the donor line PI 173437, as this may express the effect of a combination of several different resistance genes.

Race-specific resistance normally follows gene-for-gene precepts [124,125,126], and it has been confirmed that this is also valid for the Bt genes in bunt–wheat interactions [123,127,128]. When studying the virulence of the pathogen against race-specific Bt genes in wheat, it is important to take this into account. This means that infection levels in the differential set and other varieties governed by race-specific resistance genes will have a reaction either similar to a resistant line infected by an avirulent race (often close to zero) or a reaction similar to a susceptible line (often >40%), depending on environmental factors and minor race-non-specific resistance. Deviations from this binary response, therefore, indicate the influence of factors other than the direct interaction between Bt resistance genes and corresponding virulence genes.

A factor often causing problems when testing race-specific resistance is heterogeneity in either the spores or in the wheat line, causing ambiguous results where it is difficult to conclude whether the spores are virulent or avirulent to the resistance of the wheat varieties tested; it is also difficult to conclude if a given wheat variety has a resistance gene or not. If a line has a few infected plants, it cannot be finally determined whether virulence is present or not, as the infection may have been caused by other factors.

The virulence of spores to a resistant line can, in practice, only be tested by taking spores from a few infected plants of a potentially resistant variety and re-inoculating the varieties with these spores, as proposed by Flor [126]. If the infection level is higher during this test than inoculation with the original spore sample, then the small infection was caused by the presence of virulence in the spores, and the original spores were a mixture of virulent and avirulent spores. If the infection level remains at the same level, then the low infection is a sign that virulence is absent and that the wheat variety may either be genetically impure (being a mixture of resistant and susceptible lines) or the infection level is governed by race-non-specific resistance or environmental factors.

Common bunt is in practice predominantly seed-borne, but this only covers secondary infections during seed multiplication from year to year. The initial introduction of infectious spores into the crop system comes from other sources, including infection from soil [129], from spores dispersed by wind from neighbouring fields during harvesting, or from combine harvesters and other farm equipment [12,14]. This crucial distinction between primary infection and secondary spread of common bunt is important to take into account when evaluating the effect of different types of resistance mechanisms, particularly regarding race-specific versus race-non-specific resistance.

When spores are first introduced into the crop, race-specific resistance will either prevent infection or not depending on the virulence of the spores. Therefore, race-specific resistance genes will reduce the risk of primary infection to a degree relative to the proportion of spores virulent against the resistance gene. If the crop is infected by virulent spores, race-specific resistance will have no effect on subsequent multiplication in later generations. In contrast, race-non-specific resistance will reduce the level of primary infections and will also reduce the rate of secondary multiplication from year to year, but race-non-specific resistance genes will only reduce but rarely block primary infections in the pathogenesis of the disease, unless combined with genes or other control measures.

As infections increase by a factor of 100 from year to year in susceptible varieties, common bunt must be reduced by 99% by the combined control mechanisms each year to prevent this general multiplication rate. As the threshold for bunt is extremely low, a single gene of a race-non-specific resistance will rarely be able to prevent multiplication. Race-specific resistance can prevent multiplication only against avirulent spores but will have no effect against virulent spores. Therefore, a single resistance gene can rarely control common bunt alone, but a combination of genes—or resistance combined with other measures such as monitoring and discarding seed lots with primary infections—can control the disease and replace or reduce the need for fungicide applications.

## 4. Materials and Methods

### 4.1. Origin, Development, and Maintenance of Spores

Spores used in the phenotyping of germplasm in this study were originally collected in the ORGSEED project (FØJO 2001). Spores were collected from infected seed samples submitted to the Danish Plant Directorate seed health laboratory for seed health analysis. The seed samples included both certified seeds and home-saved seeds from the period 2002–2005. With such a broad material, we conclude that this material includes close to all virulence types present in Denmark during this period. The material was, at the start, not systematically tested against the differential set, but resistant varieties like ‘3540’(*Bt1*), ‘Hereward’ (*Bt_Bussard_2B* 4.1–40.9%), ‘Tommi’ (*Bt5*), Bill (*Bt5* 0.2–21.0%) and Globus (*Bt5*), ‘Trintella’ (1.8–64%), and ‘Stava’(*Bt9* + *Bt10* 0.0–1.6%) showed low infections most years; however, an increase in infections from 1999 to 2003 was seen in ‘Hereward’, ‘Bill’, and ‘Trintella’, indicating the presence of virulence [1,68,130].

To test the material for virulence, spores from infected plants of resistant varieties in the BIOBREED experiment and later follow-up research were collected and used to re-inoculate the studied variety and other varieties expected to have the same resistance gene. In cases where infection increased to the level of susceptible controls, it was concluded that virulence was present, and the spores were used in further field trials as a new race with virulence against the Bt gene. In this way, new races of common bunt were developed, in line with [75]. After a few years, races with virulence against *Bt1*, *Bt2*, *Bt3*, *Bt5*, *Bt7*, *Bt10*, and *Bt13* were developed [64,106]. Later, virulence races against *Bt4/6* and *Bt8* have been developed in the same way in the disease nursery, leaving only *Bt9* as a single Bt resistance gene without virulence.

### 4.2. Phenotyping of Germplasm

Germplasm in the current study included resistant lines from the BIOBREED project [42]; differential lines with known resistance genes demonstrated in other research; additional germplasm from gene banks, research institutions, and RILs from breeders developing bunt-resistant wheat; and our own biparental RIL populations developed to map resistance genes. Each year, differential lines and other lines with known Bt genes and other resistant varieties were included.

A total of 2731 lines were phenotyped for bunt infections by adding an excess of dry spores into paper bags with 50 seeds; they were shaken and sown by hand directly from the bags into the soil to avoid mixing of spores with different races, and sowing equipment was used. After heading, each tiller was assessed for infection by visual inspection with a focus on the development of sori in the head, and the percentage of infected tillers was recorded [131]. Tillers with partially infected heads were assessed as infected, whereas tillers expressing only leaf symptoms without sori development were recorded as healthy.

Comparing the infection level in each variety with each race, the varieties were grouped into categories with similar reactions to the different races. Varieties included in the same categories were postulated to have the same resistance gene(s). Comparing the groups with differential lines with known resistances, matching lines have been postulated to have the published Bt genes, and non-matching accessions have been postulated to have an undescribed resistance gene or a combination of resistance genes [64,106].

Based on the phenotypic reaction to different races, a collection of 1504 of the phenotyped accessions was selected for SNP genotyping using the TG26k chip Illumina Infinium by GS INSTITUT FRESENIUS GmbH (Hamburg, Germany, https://sgs-institut-fresenius.de/en/health-nutrition/traitgenetics, accessed on 27 February 2026). An additional 589 accessions were genotyped and included in the analysis based on literature information about phenotypic reactions. Supporting data on phenotypic reaction and SNP genotyping from 183 accessions on the TG15k chip was provided by NORDGEN [117] and the ECOBREED project at BOKU, Austria [55,94,96]. Phenotyping results and SNP genotyping data on the 90k chip were kindly provided by Idaho University [81,85].

### 4.3. Initial Resistance–Marker Association

For most of the Bt genes *Bt1–Bt13*, NILs with ’Red Bob’, ‘Starke-II’, or ‘Prins’ as the recurrent parent have been developed and are available in the USDA Small Grains Collection or NordGen [117,132]. In particular, NILs from ‘Starke-II’ are near-isogenic and gave relevant information as to the position of the basic Bt genes on the RefSeg v2.1 physical map [82].

Statistical analysis was based on the gene postulation based on phenotypic reactions, where each line was characterised as resistant or not towards each virulence race. Lines with a postulated resistance gene were tested against the rest of the dataset using several different mapping methods to obtain approximate locations for postulated genes: NIL mapping, GWAS, composite NIL mapping, and mappings from the literature. GWAS against gene postulates was performed with the R-package GABIT mainly using the MLM method but also GLM, FarmCPU, and Blink [118], with an FDR-corrected *p* value of 0.05 used for significance. Physical positions and chromosome assignments for marker data were obtained by BLASTing markers against the IWGSC RefSeq v2.1 [82]. Markers with positive associations with both phenotypic reactions and with a specific stable physical position were used to estimate a brutto interval within which the gene must be positioned. We consider this only as the initial step of the mapping.

### 4.4. Second Step of Gene Mapping and Development of Haplotypes for MAS

Based on the preliminary mapping of the genes, each line with a phenotypic reaction of the gene was analysed in further detail using a triplet analysis of each line with its two parents (if known). Comparing if the markers statistically significantly associated with resistance postulation were inherited from the resistant gene or from the susceptible parent, it was possible to identify the crossover events and track the origin of the haplotype interval identified in the initial mapping of the genes. Tracking recombinations of all lines, a refined mapping of the genes was assigned to the smallest interval between crossover events. Examples and further details of the description of the refined mapping procedure are presented in previous publications [113]. Missing parental information was imputed [119].

A problem when working with SNP markers is the presence of monomorphic markers between susceptible and resistant lines. A small interval gives the best position of the gene itself, but is not always optimal for MAS if it is dominated by monomorphic markers. For MAS, a selection of markers statistically best linked to resistance was chosen, and a haplotype of markers was developed that showed the closest linkage to resistance, giving the minimum of false-positive and false-negative results. Haplotypes for MAS may, in some cases, include markers outside the mapping interval of the genes, particularly in cases where markers in the mapped interval are small or dominated by monomorphic markers.

## 5. Conclusions

Differential lines have been developed to discriminate races with different virulence types, and for this purpose, differential lines may include single genes or combinations of multiple genes. However, today, differential lines are often also used to represent and define specific genes, and they are used in the mapping of the genes. For this latter purpose, differential lines should ideally only have a single resistance gene. Regarding the differential lines representing the Bt genes, we conclude that the use of the terms *Bt11*, *Bt12*, and maybe *BtP* is misleading, as the differential lines have multiple resistance genes different from other described Bt genes.

Some of the Bt genes may be identical. *Bt10* may be identical to *BtZ*, *Bt4* may be identical to *Bt6*, and one of the genes in the differential line for *Bt11* at Chr 6D may be identical to *Bt9*. Both the donors of *Bt11* and *Bt12* resistance carry resistance genes on Chr 4BS that may be identical, and similarly on Chr 4BL.

We have mapped a couple of new genes not included in the classic Bt genes, and the published literature also supports the conclusion that not only the classical Bt genes are relevant in the control of common bunt. Mappings of genes after the sequencing of the wheat genome have improved the credibility of mappings, and mappings supported by different independent studies add to this credibility. In particular, we consider nine genes as relevant candidates to evaluate in further detail for expanding and revising the differential set:

- *Bt_Mariann_1A*;
- QBt.ifa-2A (from ‘Mulan’);
- *Bt_Bussard_2B*;
- *Bt_Stephens_3A*;
- *Bt_Yayla305_5A*;
- *Bt_Yayla305_5B*;
- *Bt_Quebon_7A*;
- Qcbt.ifa-7B (from ‘Dimenit’);
- *Bt_Stephens_7B.*

More genes than these have been mapped, and in particular, in Canadian spring wheat, genes have been mapped on Chr 1BS, 1BL, 1DL, 2AL, 3DS, and 5DL. These genes, or the donors of these genes, have not been thoroughly tested in our trials, and this is also the case for *Bt14* and *Bt15*. It would be relevant to investigate these genes in further detail, including testing the potential effect of these genes in winter wheat. In addition, their specificity to a broad range of races should be tested to check their quantitative environmental dependency and race-specific behaviour.

Mapping bunt resistance genes using SNP markers is a developing work in progress, but there is a limit to how close it is possible to get to the genes using only this method. The next step is therefore to clone the genes and to develop gene-specific markers for accurate MAS.

Common bunt was, along with other seed-borne diseases, called the forgotten diseases neglected by breeding and research [18]. We believe that the research described in this paper, conducted across different institutions, countries, and continents, has returned common bunt resistance breeding to prominence as a significant tool for addressing agricultural, environmental, and human health problems related to the control of both common bunt and dwarf bunt.

## Figures and Tables

**Table 1:** Mapping of resistance.

Gene/ QTL Name	Donor Variety	Physical Position	Comment	Reference
*Bt3*	‘Ridit’Blizzard’	Chr1A: 495.06–499.90 Mbp		New mapping [107]
*Bt_Mariann_1A*	spelt	Chr1A: 1.23–10.42 Mbp		New mapping
*Bt6*	‘Rio’	Chr 1B: 16.38–28.02 Mbp		Confirmed mapping [108]
*Bt4*	PI 11610	Chr 1B: 7.48–28.02 Mbp		Confirmed mapping [109]
QBt.ifa-1B	‘Dimenit’	Chr 1B: 7.60–18.00 Mbp		New mapping
*Bt5*	‘Tommi’/‘Starke NIL-Bt5’	Chr 1B: 163.23–283.93 Mbp		Confirmed mapping [110]
*Bt2*	‘Hussar’ /PI 554097	‘Hussar’ /PI 554097		Confirmed mapping [48]
QBt.ifa-2A	‘Mulan’	Chr 2A: 0.3–35.09 Mbp		New mapping
*Bt1*	‘Martin’/PI554101	Chr 2B: 799.98–804.81 Mbp		New mapping [41]
Bt7	‘Martin’/PI554100	Chr 2D: 616.02–621.07 Mbp		Improved mapping [111]
*Bt_Stephens_3A*	‘Stephens’	Chr 3A: 683.12–688.69 Mbp		New mapping
Qbt.ifa-4BL	‘Dimenit’	Chr 4B: 657.89–662.87 Mbp	Not present in diff. line PI 554098	Improved mapping [94]
Bt_PI119333_4BL	PI 119333	Chr 4B: 650.38–670.63 Mbp		Confirmed mapping [112]
Bt_Dimenit_4BS	PI 554119/’Dimenit’	Chr 4B: 15.74–17.79 Mbp		New mapping
Bt_PI119333_4BS	PI 119333	Chr 4B: 1.31–15.86 Mbp		Confirmed mapping [112]
*Bt_Yayla305_5A*	‘Yayla 305’	Chr 5A: 572.16–597.23 Mbp		New mapping
*Bt_Yayla305_5B*	‘Yayla 305’	Chr 5B: 36.90–324.49 Mbp		New mapping
*Bt10*	PI 178383	Chr 6D: 2.05–3.34 Mbp		Improved mapping [113]
*BtZ*	‘Zarya’/‘Tilliko’	Chr 6D: 2.05–3.34 Mbp		Improved mapping [114]
*Bt9*	PI 554099	Chr 6D: 492.64–495.16 Mbp		Improved mapping [83,115]
Qbt.ifa-6DL	‘Dimenit’	Chr 6D: 492.57–492.64 Mbp		Improved mapping [94]
*Bt_Quebon_7A*	‘Quebon’/’Hereward’	Chr 7A: 671.34–676.63 Mbp.		New mapping
QBt.ifa-7AL	‘Blizzard’	Chr 7A: 717.92–735.89 Mbp		Improved mapping [55]
QCbt.ifa-7B	‘Dimenit’	Chr 7B: 10.07–12.80 Mbp		Improved mapping [94]
*Bt_Stephens_7B*	‘Stephens’	Chr 7B: 612.10–643.48 Mbp		New mapping
Bt_PI119333_7D-1	PI 119333	Chr 7D: 14.16–20.93 Mbp		New mapping
Bt_PI119333_7D-2	PI 119333	Chr 7D: 14.16–20.93 Mbp		New mapping
QCbt.ifa-7D	‘Blizzard’	Chr 7D: 16.71–21.84 Mbp	=QBt.ifa-7DS	Improved mapping [55]
*Bt13*		Chr 7D: 5.01–5.66 Mbp		Improved mapping [99]

## Data Availability

The original contributions presented in this study are included in the article. Further inquiries can be directed to the corresponding author.

## Abbreviations

The following abbreviations are used in this manuscript:

Base pair	bp
Recombinant Inbred Line	RIL
Near Isogenic Line	NIL
Mega base pair	Mbp
Genome-Wide Association Study	GWAS
Chromosome	Chr
Nomen nescio	NN

## Appendix A

*Bt1*	2B	799.991.847	803.471.859	1: Excalibur_c48404_59; Value: C 2: wsnp_Ex_c15646_23969140; Value: A 3: BS00065302_51; Value: G 4: AX-94890379; Value: G 5: BS00083998_51; Value: G 6: Ra_c105904_187; Value: C 7: Ra_c105904_1191; Value: G 8: AX-158610188; Value: A 9: AX-94808568; Value: G 10: AX-158562114; Value: C
*Bt2*	1D	41.696.687	44.673.116	1: BS00011451_51; Value: G 2: AX-94692978; Value: C 3: tplb0044p22_2330; Value: C 4: tplb0044p22_2257; Value: G 5: AX-158571869; Value: C 6: D_GBUVHFX01ANH44_230; Value: T 7: wsnp_CAP11_rep_c4017_1896951; Value: T 8: AX-94458136; Value: G
*Bt3*	1A	495.266.486	499.647.119	1: IACX5821; Value: G 2: AX-94679138; Value: C 3: AX-109834411; Value: T 4: AX-158605760; Value: T 5: AX-94632321; Value: A 6: Ra_c62804_1641; Value: C 7: Excalibur_c48379_413; Value: C 8: Excalibur_c48379_116; Value: G 9: Kukri_c27693_551; Value: G 10: Excalibur_c23992_436; Value: C 11: AX-94504536; Value: C 12: IAAV5535; Value: C 13: AX-158531952; Value: T 14: RAC875_c102223_220; Value: C 15: IACX3047; Value: G 16: AX-158537471; Value: T 17: AX-109401732; Value: A 18: AX-94462466; Value: A 19: AX-158564927; Value: T 20: AX-111506455; Value: G 21: Tdurum_contig32437_257; Value: T 22: AX-109405334; Value: T 23: IACX3595; Value: G 24: wsnp_Ex_c55986_58282517; Value: T 25: Ra_c105707_788; Value: T 26: BS00089894_51; Value: C 27: AX-158556605; Value: T 28: AX-94462123; Value: T 29: AX-158569077; Value: C 30: wsnp_Ex_rep_c109742_92411838; Value: C 31: Kukri_c73734_175; Value: C 32: AX-158536689; Value: G 33: wsnp_Ku_c21316_31053745; Value: T 34: AX-86174938; Value: G
*Bt4*	1B	7.480.290	28.018.966	1: Excalibur_c30569_384; Value: C 2: BS00005004_51; Value: T 3: AX-94668775; Value: G 4: wsnp_BE405749B_Ta_2_1; Value: C 5: Kukri_c36151_170; Value: G 6: Kukri_c44392_212; Value: G 7: BS00083562_51; Value: G 8: AX-94997422; Value: G 9: AX-94692514; Value: C 10: BS00004903_51; Value: C 11: AX-94926658; Value: A 12: Ku_c1312_1194; Value: C 13: AX-158607271; Value: G 14: BS00064829_51; Value: C 15: AX-94759371; Value: C 16: Tdurum_contig58525_304; Value: MustFail 17: AX-158540316; Value: C 18: AX-158545235; Value: MustFail 19: IAAV2147; Value: G 20: TA005766-0499; Value: G 21: AX-158570866; Value: MustFail 22: BS00108057_51; Value: MustFail 23: BS00089524_51; Value: C 24: AX-94602901; Value: C 25: BS00074962_51; Value: MustFail 26: BS00011695_51; Value: A 27: AX-108893457; Value: A 28: AX-158540300; Value: MustFail 29: AX-94570104; Value: G 30: AX-94800893; Value: MustFail 31: TGWA25K-TG0215; Value: C 32: Tdurum_contig50667_306; Value: C 33: AX-111603368; Value: MustFail 34: BS00110121_51; Value: G 35: AX-94733902; Value: G 36: Excalibur_c3270_1566; Value: A 37: Excalibur_c3270_1566; Value: A
*Bt5*	1B	164.434.858	271.683.038	1: BS00066165_51; Value: G 2: BS00022625_51; Value: G 3: BS00106581_51; Value: G 4: AX-158556915; Value: A 5: AX-110422876; Value: C 6: AX-95248610; Value: A 7: RAC875_rep_c72356_51; Value: C 8: AX-158555575; Value: T 9: Kukri_c19709_383; Value: T 10: AX-158561091; Value: C 11: wsnp_BE637864B_Ta_1_1; Value: G 12: AX-158540267; Value: C 13: Kukri_c147_1620; Value: G 14: BS00022317_51; Value: A 15: AX-158561110; Value: G 16: AX-158570854; Value: C 17: AX-110035800; Value: A 18: Kukri_rep_c105316_262; Value: T 19: AX-158520903; Value: G 20: AX-109447012; Value: C 21: BS00091191_51; Value: G 22: AX-108921445; Value: G 23: AX-158540090; Value: A 24: AX-111514779; Value: C
*Bt6*	1B	16.466.442	25.179.826	1: BS00011695_51; Value: A 2: AX-108893457; Value: A 3: AX-158540300; Value: MustFail 4: AX-94570104; Value: G 5: AX-94800893; Value: MustFail 6: TGWA25K-TG0215; Value: C 7: Tdurum_contig50667_306; Value: C 8: AX-111603368; Value: MustFail 9: BS00110121_51; Value: G 10: AX-94733902; Value: G 11: Excalibur_c3270_1566; Value: A
*Bt7*	2D	621.068.206	622.542.240	1: RAC875_c30919_311; Value: G 2: RAC875_rep_c114621_200; Value: C 3: wsnp_Ex_c42970_49408712; Value: A
*Bt8*	4A	1.887.532	16.857.414	1: AX-94471577; Value: C 2: AX-158542180; Value: A 3: BS00021716_51; Value: C 4: BS00106545_51; Value: A 5: AX-108808218; Value: G 6: AX-158542161; Value: C 7: BS00043286_51; Value: G 8: wsnp_Ex_c14478_22481430; Value: C 9: AX-94381780; Value: T 10: BS00108852_51; Value: T 11: AX-111488229; Value: G 12: AX-95104288; Value: C 13: BS00065863_51; Value: T 14: AX-94715337; Value: A 15: AX-94409394; Value: C 16: wsnp_BE405275A_Ta_1_1; Value: G 17: wsnp_RFL_Contig2771_2524880; Value: G 18: AX-94514459; Value: G 19: AX-89419039; Value: C 20: AX-158524876; Value: C 21: wsnp_Ex_c28429_37553452; Value: A 22: AX-158542163; Value: C
*Bt9*	6D	494.217.075	495.158.655	1: AX-109985406; Value: T 2: RAC875_rep_c104893_620; Value: C 3: RAC875_c2910_1562; Value: G 4: AX-109853614; Value: A 5: BS00070856_51; Value: T 6: RFL_Contig2615_982; Value: T 7: RFL_Contig2615_700; Value: A 8: AX-94841369; Value: A 9: AX-94747666; Value: T 10: AX-109917993; Value: C
*Bt10*	6D	2.388.914	3.140.478	2: Kukri_c55362_75; Value: A 3: AX-108746724; Value: C 4: Excalibur_c7731_2743; Value: A 5: AX-158531240; Value: C 6: wsnp_Ex_c18664_27540364; Value: G
*Bt13*	7D	5.542.839	7.432.654	1: Ra_c30952_531; Value: T 2: AX-158544378; Value: T 3: AX-94708419; Value: G 4: TA001746-1415; Value: G 5: Kukri_c80931_147; Value: A
*BtZ*	6D	2.388.914	3.140.478	1: wsnp_Ex_c14439_22426200; Value: T 2: Kukri_c55362_75; Value: C 3: AX-108746724; Value: C 4: Excalibur_c7731_2743; Value: G 5: AX-158531240; Value: T
Bt_Blizzard_7A	7A	623.904.095	728.815.254	1: IAAV5550; Value: C 2: BS00084193_51; Value: C 3: Kukri_c28968_130; Value: G 4: BS00105531_51; Value: C 5: BS00027226_51; Value: A 6: BS00002510_51; Value: T 7: BS00067682_51; Value: C 8: BS00068033_51; Value: C 9: Kukri_c51453_406; Value: T
Bt_Blizzard_7D	7D	16.714.316	21.837.392	1: RAC875_rep_c104791_336; Value: G 2: Kukri_c20949_503; Value: T 3: AX-95229555; Value: T 4: Kukri_c12113_837; Value: A 5: AX-94411546; Value: T 6: AX-158601965; Value: C 7: BobWhite_c20306_88; Value: A 8: BobWhite_c11327_185; Value: T 9: BobWhite_c11327_248; Value: G 10: TA005893-0466; Value: G 11: BobWhite_c30138_69; Value: T 12: GENE-4277_295; Value: C 13: Excalibur_c1310_414; Value: G 14: Excalibur_c16355_712; Value: T 15: AX-158559855; Value: T
Bt_Dimenit_6D_1	6D	494.217.075	495.158.655	1: AX-109985406; Value: C 2: RAC875_rep_c104893_620; Value: C 3: RAC875_c2910_1562; Value: G 4: AX-109853614; Value: G 5: BS00070856_51; Value: G 6: RFL_Contig2615_982; Value: C 7: RFL_Contig2615_700; Value: A 8: AX-94841369; Value: T 9: AX-94747666; Value: T 10: AX-109917993; Value: T
Bt_Dimenit_6D_2	6D	494.217.075	495.158.655	1: AX-109985406; Value: C 2: RAC875_rep_c104893_620; Value: T 3: RAC875_c2910_1562; Value: A 4: AX-109853614; Value: G 5: BS00070856_51; Value: G 6: RFL_Contig2615_982; Value: C 7: RFL_Contig2615_700; Value: G 8: AX-94841369; Value: T 9: AX-94747666; Value: T 10: AX-109917993; Value: T
Bt_PI119333_7D	7D	14.163.614	20.292.538	1: Excalibur_c833_1405; Value: G 2: AX-94735600; Value: G 3: AX-111070966; Value: A 4: Ex_c25027_535; Value: T 5: wsnp_Ra_c8297_14095831; Value: T 6: BS00110642_51; Value: T 7: AX-94930280; Value: A 8: AX-94398131; Value: A 9: BobWhite_c8454_782; Value: G 10: RAC875_rep_c104791_336; Value: G 11: Kukri_c20949_503; Value: T 12: AX-95229555; Value: T 13: Kukri_c12113_837; Value: A 14: AX-94411546; Value: T 15: AX-158601965; Value: C 16: BobWhite_c20306_88; Value: A 17: BobWhite_c11327_185; Value: T 18: BobWhite_c11327_248; Value: G
Bt_PI119333_4BS	4B	1.276.289	15.848.436	1: AX-158564576; Value: C 2: Tdurum_contig81460_347; Value: T 3: AX-94572741; Value: G 4: AX-109946437; Value: G 5: Tdurum_contig11733_825; Value: T 6: AX-158538742; Value: A 7: BS00039935_51; Value: G 8: BS00039936_51; Value: A 9: BS00063809_51; Value: G 10: AX-158542337; Value: T 11: AX-95190182; Value: G 12: AX-158598944; Value: A 13: BS00060041_51; Value: T 14: AX-95143067; Value: T 15: AX-158538739; Value: A 16: AX-158542312; Value: A 17: AX-109388531; Value: A 18: AX-158538740; Value: A 19: AX-111481149; Value: A 20: AX-158564641; Value: A 21: AX-158583365; Value: A 22: AX-158542333; Value: C 23: AX-89538793; Value: G 24: Tdurum_contig10322_1908; Value: A 25: wsnp_Ra_c9755_16200944; Value: T 26: Tdurum_contig93710_409; Value: A 27: BS00037094_51; Value: A 28: AX-158564494; Value: G 29: AX-158583339; Value: G 30: AX-158542402; Value: C 31: Tdurum_contig47622_234; Value: A 32: AX-110907280; Value: T 33: Tdurum_contig67399_676; Value: A 34: AX-110382283; Value: C 35: AX-158542410; Value: G 36: Tdurum_contig76559_124; Value: G
Bt_PI119333_4BL	4B	651.349.625	673.432.269	1: Tdurum_contig9893_492; Value: C 2: RAC875_c51375_394; Value: C 3: AX-94448564; Value: G 4: AX-95258779; Value: G 5: AX-109865770; Value: T 6: AX-110958367; Value: T 7: BobWhite_c4256_213; Value: A 8: AX-158598904; Value: C 9: BS00104279_51; Value: T 10: wsnp_Ex_c4148_7494801; Value: T 11: AX-111537772; Value: G 12: BS00034148_51; Value: T 13: AX-111607064; Value: T 14: AX-158550172; Value: A 15: AX-94492644; Value: C 16: AX-158582661; Value: C 17: AX-94433424; Value: G 18: AX-158550140; Value: C
Bt_Mulan_2A	2A	259.719	34.967.976	20: Tdurum_contig29983_490; Value: C 21: AX-109964399; Value: C 22: Ex_c19516_3687; Value: C 23: RFL_Contig174_406; Value: A 24: AX-158573559; Value: G 25: BobWhite_c13373_250; Value: G 26: TGWA25K-TG0117; Value: T 27: TG0117; Value: T 28: AX-94446514; Value: A 29: wsnp_Ex_c11950_19164191; Value: T 30: AX-94956032; Value: G 31: wsnp_Ku_c33374_42877546; Value: C 32: Kukri_c33374_1048; Value: T 33: tplb0032i02_1388; Value: C 34: Excalibur_c12980_2621; Value: A 35: Excalibur_c12980_2392; Value: A 36: RAC875_c42847_141; Value: T 37: IAAV8501; Value: T 38: RAC875_c2300_1021; Value: A 39: RAC875_c63883_76; Value: A 40: BS00022760_51; Value: C 41: AX-94944993; Value: G 42: IACX6178; Value: C 43: CAP12_c259_307; Value: T 44: AX-94679104; Value: C 45: AX-94906650; Value: C 46: RAC875_c829_1143; Value: C 47: RAC875_c829_355; Value: T 48: IACX11417; Value: A 49: Kukri_c29358_277; Value: T 50: BS00039973_51; Value: T 51: BS00021706_51; Value: A 52: AX-95126447; Value: G 53: TA003766-0683; Value: G 54: BS00093990_51; Value: C 55: AX-158540813; Value: T 56: RAC875_rep_c111906_144; Value: A 57: AX-94381659; Value: T 58: AX-94717890; Value: A 59: CAP8_rep_c8022_270; Value: C 60: BobWhite_c2022_245; Value: G 61: CAP11_c2293_200; Value: G 62: wsnp_Ex_c61879_61748626; Value: A
Bt_Dimenit_4BS	4B	15.848.436	17.149.339	1: Tdurum_contig76559_124; Value: G 2: Excalibur_c7581_791; Value: C 3: Excalibur_rep_c79414_306; Value: A 4: AX-110579601; Value: G 5: Tdurum_contig76213_958; Value: C 6: Tdurum_contig82942_681; Value: G
Bt_Dimenit_4BL	4B	658.074.570	662.257.892	1: TA003210-1094; Value: A 2: wsnp_BE403378B_Ta_2_1; Value: C 3: BS00027054_51; Value: G
Bt_Quebon_7A	7A	656.861.151	679.827.267	1: AX-94463677; Value: A 2: AX-158566892; Value: T 3: AX-158566889; Value: T 4: AX-94721829; Value: C 5: Excalibur_c84687_162; Value: G 6: RAC875_c20121_561; Value: A 7: AX-94531661; Value: T 8: AX-158590659; Value: A 9: AX-158589980; Value: A 10: BobWhite_c1215_240; Value: G 11: AX-158567102; Value: C 12: BS00088825_51; Value: G 13: BobWhite_c15352_394; Value: G 14: BS00071478_51; Value: T 15: AX-158553168; Value: C 16: RAC875_c37085_317; Value: A 17: Kukri_c24408_743; Value: T 18: JD_c149_1700; Value: T 19: BobWhite_c12302_389; Value: T 20: Ra_c14761_1348; Value: T 21: wsnp_Ku_c42539_50247333; Value: C 22: BobWhite_c1201_384; Value: T 23: wsnp_Ku_c42539_50247426; Value: A 24: AX-158567056; Value: A 25: BS00023128_51; Value: A 26: AX-158543577; Value: G 27: wsnp_Ex_c9428_15641609; Value: C 28: wsnp_Ex_c9428_15641639; Value: A 29: AX-158556233; Value: T 30: BS00021657_51; Value: T 31: AX-158559587; Value: A 32: AX-158625860; Value: G 33: Excalibur_c95707_285; Value: T 34: AX-158556230; Value: G 35: BS00026622_51; Value: G 36: AX-158537273; Value: C 37: wsnp_JD_c20555_18262317; Value: A 38: RAC875_c19111_628; Value: C 39: AX-108837168; Value: A 40: AX-94439426; Value: G 41: IAAV6957; Value: G 42: tplb0045p11_893; Value: T
Bt_Bussard_2B	2B	8.697.088	13.124.351	1: RAC875_rep_c71112_400; Value: A 2: Excalibur_c34937_710; Value: T 3: AX-94831339; Value: C 4: AX-94880001; Value: A 5: AX-109959677; Value: A 6: BS00023068_51; Value: C 7: AX-158562561; Value: T 8: BS00044332_51; Value: C 9: BS00084668_51; Value: A 10: wsnp_Ex_c1996_3754394; Value: T 11: RAC875_rep_c115433_378; Value: C 12: AX-94505732; Value: T 13: TGWA25K-TG0159; Value: A
Bt_Mariann_1A	1A	1.208.845	10.424.272	1: RAC875_c95364_259; Value: A 2: Tdurum_contig44888_837; Value: C 3: BS00033749_51; Value: G 4: BS00026456_51; Value: T 5: AX-89562713; Value: G 6: TGWA25K-TG0108; Value: T 7: TG0108; Value: T 8: Ku_c28007_1398; Value: A 9: AX-111569969; Value: A 10: AX-158555547; Value: A 11: AX-158560734; Value: C 12: AX-158569633; Value: G 13: BS00073243_51; Value: T 14: BS00023201_51; Value: G 15: BS00022355_51; Value: C 16: AX-110068701; Value: C 17: wsnp_Ex_c57982_59470152; Value: A 18: AX-110004070; Value: C
Bt_Stephens_3A	3A	683.220.645	688.690.639	1: RAC875_c10194_673; Value: C 2: AX-95132491; Value: A 3: Excalibur_c29600_173; Value: C 4: AX-158533015; Value: G 5: wsnp_Ex_c1894_3575749; Value: G 6: RAC875_c15003_377; Value: C 7: BS00063696_51; Value: A 8: Kukri_c8465_54; Value: G 9: AX-89724344; Value: A 10: AX-109295307; Value: T 11: BS00039498_51; Value: G 12: wsnp_Ex_c12341_19693570; Value: G 13: wsnp_Ex_c12341_19693090; Value: A 14: AX-108911182; Value: T 15: AX-158538215; Value: A 16: wsnp_Ex_c27317_36522052; Value: C 17: wsnp_CAP11_rep_c4226_1995152; Value: G 18: IAAV1410; Value: T 19: IAAV5370; Value: A 20: wsnp_Ex_rep_c66357_64540428; Value: T 21: Ex_c66357_866; Value: C 22: wsnp_Ra_c132_291198; Value: G 23: wsnp_Ex_rep_c66357_64540369; Value: T 24: Kukri_c12079_204; Value: A 25: Tdurum_contig59585_656; Value: A 26: wsnp_JD_c29019_23208279; Value: A 27: AX-158538218; Value: T 28: AX-158538236; Value: T 29: BS00081610_51; Value: T 30: AX-95003297; Value: C 31: BS00023337_51; Value: G 32: BS00060029_51; Value: A 33: BS00088756_51; Value: C 34: BS00088755_51; Value: T 35: AX-86168015; Value: G 36: BobWhite_c5337_225; Value: T 37: AX-158523254; Value: G
Bt_Stephens_7B	7B	612.396.641	643.412.748	1: AX-158554033; Value: G 2: AX-94676341; Value: C 3: RAC875_c4834_694; Value: T 4: AX-158554039; Value: G 5: AX-94505411; Value: T 6: Ku_c5351_1820; Value: G 7: BobWhite_rep_c66630_331; Value: C 8: AX-158592651; Value: C 9: BobWhite_c12256_96; Value: T 10: AX-158567766; Value: C 11: AX-158592634; Value: A 12: AX-108870188; Value: T 13: AX-89599935; Value: C 14: BS00022045_51; Value: C 15: wsnp_Ex_c10550_17231294; Value: C 16: AX-110432367; Value: A 17: RAC875_c27548_417; Value: G 18: BS00089942_51; Value: G 19: RAC875_c27548_234; Value: T 20: AX-158592661; Value: G 21: wsnp_Ku_c17161_26193994; Value: A 22: wsnp_Ku_c17161_26193672; Value: T 23: GENE-4624_79; Value: C 24: AX-158567774; Value: A 25: Kukri_c51101_351; Value: T 26: RAC875_c24101_284; Value: C 27: AX-158544029; Value: C 28: AX-95021316; Value: C 29: AX-110369629; Value: G 30: RAC875_c21489_908; Value: C 31: IACX486; Value: C 32: AX-158591808; Value: C
Bt_Yayla305_5A	5A	572.654.222	597.231.033	1: AX-94406443; Value: A 2: Tdurum_contig71499_211; Value: G 3: wsnp_Ex_c7266_12475249; Value: C 4: wsnp_Ex_c1138_2185522; Value: A 5: AX-158584923; Value: G 6: AX-158542656; Value: C 7: Excalibur_c45297_316; Value: A 8: AX-109433082; Value: T 9: wsnp_Ex_rep_c69647_68598463; Value: G 10: AX-158620334; Value: G 11: AX-158558755; Value: A 12: Tdurum_contig82190_124; Value: C 13: AX-94432465; Value: C 14: AX-109331427; Value: T 15: AX-158551020; Value: C 16: Tdurum_contig44343_1039; Value: G 17: AX-158542740; Value: C 18: AX-158551088; Value: G 19: AX-108744896; Value: C 20: AX-110426237; Value: C 21: Ku_c19858_2078; Value: C 22: AX-94619088; Value: A 23: BS00076246_51; Value: C 24: Tdurum_contig86202_145; Value: C 25: Tdurum_contig86202_175; Value: A 26: BobWhite_c23736_153; Value: A 27: Tdurum_contig52695_388; Value: T 28: AX-109849058; Value: A 29: AX-89764932; Value: G 30: GENE-3601_145; Value: C 31: AX-94837642; Value: T 32: AX-158550911; Value: C 33: AX-158565171; Value: T 34: BS00044408_51; Value: C 35: AX-89311025; Value: T 36: wsnp_Ku_c20011_29589514; Value: T 37: wsnp_Ku_c20011_29589289; Value: A 38: wsnp_Ku_c20011_29589089; Value: G 39: Excalibur_c37943_221; Value: T 40: AX-158550736; Value: A 41: AX-95629509; Value: C 42: wsnp_Ra_c12183_19587379; Value: G 43: Kukri_c29560_455; Value: G 44: BS00065481_51; Value: T 45: AX-158584285; Value: G 46: AX-111040754; Value: G 47: AX-158584445; Value: T 48: AX-94442743; Value: G 49: AX-111483425; Value: G 50: AX-158538907; Value: G 51: AX-158538950; Value: A 52: AX-111072968; Value: A 53: RAC875_c86041_91; Value: C 54: TG0053; Value: C 55: TGWA25K-TG0053; Value: C 56: TG0020; Value: C 57: TGWA25K-TG0041; Value: G 58: TG0041; Value: G 59: BS00075959_51; Value: A 60: AX-158584403; Value: G 61: AX-158542533; Value: A 62: BS00088851_51; Value: C 63: CAP11_c3209_76; Value: A 64: AX-158585018; Value: C 65: AX-94391667; Value: A 66: AX-158584526; Value: T 67: AX-109435061; Value: A
Bt_Yayla305_5B	5B	48.668.280	284.765.351	1: AX-158534310; Value: A 2: AX-158526437; Value: T 3: AX-94612603; Value: G 4: Tdurum_contig25068_259; Value: T 5: Kukri_s113060_116; Value: C 6: AX-158526315; Value: A 7: AX-94691166; Value: G 8: AX-94467784; Value: T 9: wsnp_Ku_c7872_13484038; Value: G 10: Ex_c2571_987; Value: G 11: wsnp_Ex_c58012_59490259; Value: C 12: GENE-0782_747; Value: A 13: BS00074315_51; Value: G 14: JD_c16284_736; Value: C 15: BobWhite_c4852_323; Value: G 16: AX-110419826; Value: C 17: TA006084-0922; Value: A 18: wsnp_Ku_c32477_42087329; Value: G 19: wsnp_Ex_rep_c104986_89538820; Value: C 20: BS00067028_51; Value: G 21: wsnp_Ra_c5210_9289264; Value: T 22: wsnp_BQ166999B_Ta_2_1; Value: T 23: Kukri_c13224_551; Value: A 24: wsnp_Ex_c12431_19823475; Value: G 25: wsnp_Ex_c24577_33826666; Value: G 26: AX-158526448; Value: G 27: RAC875_c96137_101; Value: C 28: Kukri_c40388_844; Value: A 29: IAAV8999; Value: A 30: IAAV4590; Value: G 31: wsnp_Ex_c18519_27369737; Value: C 32: AX-158565742; Value: C 33: AX-158526336; Value: C 34: AX-158525931; Value: C 35: Excalibur_c97201_294; Value: G 36: wsnp_Ex_c658_1294003; Value: G 37: AX-158539087; Value: C 38: AX-158534082; Value: G 39: AX-158533793; Value: C 40: wsnp_CAP7_c2086_1018815; Value: G 41: BobWhite_c36054_53; Value: A 42: wsnp_Ex_c39535_46808105; Value: G 43: Kukri_c31961_630; Value: T

## Appendix B

Mapping of resistances compared with references

**Gene/QTL** **Name**	**Donor Variety**	**Physical Position**	**Comment**	**Reference**
*Bt3*	‘Ridit’/‘Blizzard’	Chr1A: 495.06–499.90 Mbp		New mapping [107]
Q.DB.ui-1A	‘IDO444’	Chr1A: 503.31 Mbp	Possibly *Bt3*	[44,97]
Qbt.ifa-1A	‘Dimenit’	Chr1A: 355.2–515.2 Mbp	Possibly *Bt3*	[94]
NN	diversity panel	Chr1A: 473.97 Mbp	Possibly *Bt3*	[44]
Qbt.ifa-1AL	‘Blizzard’	Chr1A: 498.5–516.6 Mbp	Possibly *Bt3*	[55,56]
NN	diversity panel	Chr1A: 497.93–499.86 Mbp	Possibly *Bt3*	[43]
QCbt.dms-1A.3	diversity panel	Chr1A: 556.87 Mbp	Possibly *Bt3*	[90]
QCbt.dms-1A.2	diversity panel	Chr1A: 13.37–14.03 Mbp		[90]
QCbt.dms-1A.1	diversity panel	Chr1A: 4.38 Mbp		[90]
Bt_Mariann_1A	spelt	Chr1A: 1.23–10.42 Mbp		New mapping
*Bt6*	‘Rio’	Chr 1B: 16.38–28.02 Mbp		Confirmed mapping [108]
*Bt4*	PI 11610	Chr 1B: 7.48–28.02 Mbp		Confirmed mapping [109]
QBt.ifa-1B	‘Dimenit’	Chr 1B: 7.60–18.00 Mbp	possibly *Bt6*	New mapping
NN	‘Blizzard’	Chr 1B: 8–22 Mbp	possibly *Bt6*	[55]
QBt.ifa-1BS	‘Dimenit’	Chr 1B: 2.2 and 46.9 Mbp	possibly *Bt6*	[94]
NN	diversity panel	Chr 1B: 11.18 Mbp	possibly *Bt6*	[44]
NN	‘Blizzard’	Xgwm374 Xbarc128 and Xgwm264	possibly *Bt6*	[70]
*Bt5*	‘Tommi’/‘Starke NIL-Bt5’	Chr 1B: 163.23–283.93 Mbp		Confirmed mapping [110]
NN	diversity panel	Chr 1B: 137.13–163.10 Mbp	possibly *Bt5*	[43]
QCbt.dms-1B.2	‘CDC Go’	Chr 1B: 551.90–517.23 Mbp	Minor effect gene	[73]
QCbt.dms-1B	‘Carberry’	Chr 1B: 21.4 Mb	Minor effect gene	[46]
QCbt.dms-1B	diversity panel	Chr 1B: 21.0–21.4 Mbp	Minor effect gene	[90]
QCbt.spa-1B	‘Carberry’	Flanking: wPt-667763–wPt-731722 (=517.23 Mbp)	Minor effect gene	[72]
Q Cbt.crc-1B.2	‘AC Domain’	Xgwm403	Minor effect gene	[71]
Q Cbt.crc-1B.1	‘AC Domain’	Xgwm374.1 (173.53 Mbp) and Xwmc818b	Minor effect gene	[71]
NN	‘Trintella’	45 cM Xgwm273 near the centromere (Xgwm273 = WMS273: 218.88 Mbp)	Minor effect gene	[69]
*Bt2*	‘Hussar’ /PI 554097	Chr 1D: 41.70–44.67 Mbp		Confirmed mapping [48]
QCbt.spa-1D	‘Vesper’	Chr 1D: 595.40 Mbp	Position uncertain	[49]
QBt.ifa-2A	‘Mulan’	Chr 2A: 0.3–35.09 Mbp		New mapping
QBt.ifa-2A	‘Mulan’*‘Dimenit’	Chr 2A: 259.75–Mbp	Tdurum_contig29983_490 and AX-94381641	[94]
QCbt.spa-2A	‘Vesper’	Chr 2A: 745.40–746.74 Mbp		[49]
*Bt1*	‘Martin’/PI554100	Chr 2B: 799.98–804.81 Mbp		New mapping [41]
NN	diversity panel	Chr 2B: 787.82–785.91 Mbp	Possibly *Bt1*	[43]
NN	diversity panel	Chr 2B: 581.70 Mbp	Possibly *Bt1*	[44]
QCbt.cph-2B	diversity panel	Chr 2B: 655–1591 Mbp	Possibly *Bt1* (position according to reference)	[42]
Bt_Bussard_2B	‘Bussard’	Chr 2B: 8.70–13.12 Mbp	Phenotypical identical to *Bt2*	[48]
QCbt.dms-2B	‘CDC Go’	Chr 2B: 244.0 Mb		[46]
Q.DB.ui-2B	‘IDO444’	Chr 2B: Peak: Xwmc317 14 cM		[97]
Bt7	‘Martin’/PI554100	Chr 2D: 616.02–621.07 Mbp		Improved mapping [111]
NN	‘Lewjain’	wmc112	Position uncertain	[79]
Bt_Stephens_3A	‘Stephens’	Chr 3A: 683.12–688.69 Mbp		New mapping
QCbt.dms-3A.2	diversity panel	Chr 3A: 671.29 Mbp		[90]
NN	diversity panel	Chr 3A: 69.957–742.47 Mbp		[43]
NN	diversity panel	Chr 3A: 51.29–53.74 Mbp		[43]
QCbt.dms-3A.1	diversity panel	Chr 3A: 10.28 Mbp		[90]
QCbt.dms- 3A	‘CDC Go’	Chr 3A: 1.48–251.80 Mbp	Not confirmed in a later study [46]	[73]
NN	diversity panel	Chr 3B: 0.85–6.95 Mbp		[43]
QCbt.spa-3D	‘Lillian’	Chr 3D: 3.12–3.98 Mbp		[49]
Qbt.ifa-4BL	‘Dimenit’	Chr 4B: 657.89–662.87 Mbp	Not present in diff. line PI 554098	Improved mapping
Qbt.ifa-4BL	‘Dimenit’	Chr 4B: 662.9 and 671.4 Mbp		[94]
Bt_PI119333_4BL	PI 119333	Chr 4B: 650.38–670.63 Mbp		Confirmed mapping [112]
Bt_Dimenit_4BS	PI 554119/’Dimenit’	Chr 4B: 15.74–17.79 Mbp		New mapping
NN	‘Dimenit’	not specified	different from Qbt.ifa-4BL	[94]
Bt_PI119333_4BS	PI 119333	Chr 4B: 1.31–15.86 Mbp		Confirmed mapping [112]
QBt.ifa-4B	PI 119333	Chr 4B: 20.6–706.5 Mbp		[96]
QCbt.spa-4B	‘Carberry’	Position uncertain (167.48 Mbp)	flanking: wPt-744434–wPt-617	[72]
Bt_Yayla305_5A	‘Yayla 305’	Chr 5A: 572.16–597.23 Mbp		New mapping
	diversity panel	Chr 5A: 568.05–613.55 Mbp		[43]
Qcbt.spa-5A	‘Lillian’	Chr 5A: 659.01–658.82 Mbp		[49]
Bt_Yayla305_5B	‘Yayla 305’	Chr 5B: 36.90–324.49 Mbp		New mapping
NN	‘Trintella’	Chr 5B: 0–19 cM, nearest marker Xgwm408		[69]
NN		Chr 5D: 544.28–545.10 Mbp		[43]
QCbt.dms-5D.1	diversity panel	Chr 5D: 244.10 Mbp		[90]
QCbt.dms-5D.2	diversity panel	Chr 5D: 565.87 Mbp		[90]
NN	diversity panel	Chr 6A: 431.92–611.86 Mbp		[43]
NN	‘Kenyon’	Chr 6A: Not specified		[45]
NN	diversity panel	Chr 6B: 461.41–708.26 Mbp		[43]
*Bt10*	PI 178383	Chr 6D: 2.05–3.34 Mbp		Improved mapping [113]
*BtZ*	‘Zarya’/’Tilliko’	Chr 6D: 2.05–3.34 Mbp		Improved mapping [114]
QCbt.dms-6D	‘Peace’	Chr 6D: 7.6 Mb	*=Bt10*	[46]
QCbt.dms-6D	diversity panel	Chr 6D: 7.43 Mbp	*=Bt10*	[90]
QCbt.spa-6D	‘AC Cadillac’	Flanking: wPt-672044–wPt-5114 (=6.17 Mbp)	*=Bt10*	[72]
Qdb.ssdhui-6DS	‘UI Silver’	Chr 6D: 1.4–2.1 Mbp	*=Bt10*	[85]
DB-6D1	‘IDO835’	Chr 6D: 1.77 Mbp	*=Bt10*	[84]
DB-6D2	‘IDO835’	Chr 6D: 6.97 to 7.29 Mbp	*=Bt10*	[84]
Qdb.ssdhui-6DL	‘UI Silver’	Chr 6D: 492.5–494.6 Mbp	*=Bt9*	[85]
*Bt9*	PI 554099	Chr 6D: 492.64–495.16 Mbp		Improved mapping [83,115]
Qbt.ifa-6DL	‘Dimenit’	Chr 6D: 482.8–495.2 Mbp		[94]
Qbt.ifa-6DL	‘Dimenit’	Chr 6D: 492.57–492.64 Mbp		Improved mapping
Bt_Quebon_7A	‘Quebon’/’Hereward’	Chr 7A: 671.34–676.63 Mbp.		New mapping
QBt.ifa-7AL	‘Blizzard’	Chr 7A: 717.92–735.89 Mbp		Improved mapping
QBt.ifa-7AL	‘Blizzard’	Chr 7A: 722–737 Mbp		[55]
QDB.ui-7AL	‘IDO835’	Chr 7A: 732.05–736.57 Mbp		[81]
QCbt.spa-7A	‘Lillian’	Chr 7A: 598.87–693.40 Mbp		[49]
Q Cbt.crc-7A	‘AC Domain’	Chr 7A: 686.3–688.6 Mbp		[71]
QCbt.cph-7A,	diversity panel	Chr 7A: 444.4 Mbp		[42]
NN	diversity panel	Chr 7A: 335.99 Mbp		[44]
NN	diversity panel	Chr 7A: 633.77 Mbp	Position and Chr uncertain	[44]
NN	‘Trintella’	Uncertain position	32.7–48.5 cM nearest marker Xpsp3050	[69]
NN	diversity panel	Chr 7A: 298.99–298.99 Mbp		[43]
QCbt.ifa-7B	‘Dimenit’	Chr 7B: 10.07–12.80 Mbp		Improved mapping
QBt.ifa-7B	‘Dimenit’	Chr 7B: 7.1–26.4 Mbp		[94]
NN	diversity panel	Chr 7B: 18.1 Mbp		[43]
Bt_Stephens_7B	‘Stephens’	Chr 7B: 612.10–643.48 Mbp		New mapping
NN	diversity panel	Chr 7B: 703.15 Mbp		[43]
QCbt.spa-7B.1	‘McKenzie’	Position uncertain	Peak: Xgwm573 and Xwmc17	[95]
NN	‘Trintella’	Chr 7B: 417–544 Mbp		[69]
Bt_PI119333_7D-1	PI 119333	Chr 7D: 14.16–20.93 Mbp		New mapping
Bt_PI119333_7D-2	PI 119333	Chr 7D: 14.16–20.93 Mbp		New mapping
QBt.ifa-7DS	PI 119333	Chr 7D: 6.47–10.84 Mbp		[96]
QCbt.ifa-7D	‘Blizzard’	Chr 7D: 16.71–21.84 Mbp	=QBt.ifa-7DS	Improved mapping
Q.DB.ui-7DS	‘IDO444’	Chr 7D: 5.88 Mbp	=QBt.ifa-7DS	[97]
QBt.ifa-7DS	‘Blizzard’	Chr 7D: 12.5–15.3 Mbp	=Q.DB.ui-7DS	[55]
*Bt13*		Chr 7D: 5.01–5.66 Mbp		Improved mapping [99]
